# Under‐Urine‐Adhered Supramolecular Hydrogel with Linearly Sustained Quercetin Release Facilitates Hemorrhagic Cystitis Healing via Inflammation Regulation

**DOI:** 10.1002/advs.202515003

**Published:** 2025-11-07

**Authors:** Xu Cao, Hua Zhang, Yang Luo, Yaoqi Chen, Jie Yao, Renhao Ni, Tong Zhu, Yudong Yao, Jun Chen, Baolin Guo, Kerong Wu

**Affiliations:** ^1^ Department of Urology Translational Research Laboratory for Urology Ningbo Clinical Research Center for Urological Disease The First Affiliated Hospital of Ningbo University Zhejiang Engineering Research Center of Innovative Technologies and Diagnostic and Therapeutic Equipment for Urinary System Diseases Ningbo Zhejiang 315010 China; ^2^ Research Institute of Smart Medicine and Biological Engineering Health Science Center Ningbo University Ningbo Zhejiang 315211 China; ^3^ Intelligent Polymer Research Institute Innovation Campus University of Wollongong Squires Way North Wollongong NSW 2500 Australia; ^4^ State Key Laboratory for Mechanical Behavior of Materials and Frontier Institute of Science and Technology Xi'an Jiaotong University Xi'an Shaanxi 710049 China

**Keywords:** anti‐inflammation, hemorrhagic cystitis, hydrogel adhesives, quercetin release

## Abstract

The clinical management of hemorrhagic cystitis remains challenging because of persistent inflammation and bladder mucosal barrier disruption. Although intravesical therapies, such as quercetin and hyaluronic acid (HA), show potential, their efficacy is limited by rapid urinary clearance. Hydrogels offer potential as sustained‐release drug carriers and protective barriers, yet designing adhesive hydrogels with optimal wet tissue adhesion, controlled degradation, and regulation of anti‐inflammation remains difficult. Here it is aimed to develop a supramolecular gelatin‐based hydrogel adhesive (HADA/gelatin/Ac‐β‐CD/quercetin, HGCQ) that combines acrylated β‐cyclodextrin as a dynamic crosslinker and hydrophobic drug carrier with dopamine‐functionalized HA for enhanced tissue adhesion and antioxidant functionality. The HGCQ hydrogel exhibits controlled degradation in artificial urine with sustained quercetin release over 48 h, exceptional burst pressure tolerance of up to 18.8 kPa on the bladder, and rapid hemostatic capability with a clotting time of 15 s. Crucially, HGCQ demonstrates comprehensive therapeutic effects including pro‐angiogenic potential, and broad‐spectrum antimicrobial efficacy, potent reactive oxygen species scavenging with a clearance of 600 µm H_2_O_2_, and anti‐inflammatory activity mediated by nuculear factor kappa‐B (NF‐κB)/interleukin (IL)‐17 pathways. In hemorrhagic cystitis models, HGCQ restores urothelial barrier function, promotes collagen III deposition, and reduces inflammation, making a significant advancement in managing this condition with potential applications in other wound healing scenarios.

## Graphical abstract

A supramolecular gelatin‐based hydrogel adhesive was engineered for hemorrhagic cystitis management. It provides robust wet‐tissue adhesion, rapid hemostasis, sustained quercetin release, effectively restoring the bladder barrier via anti‐inflammatory and antioxidant actions.

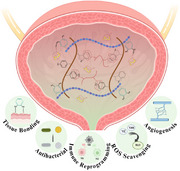

## 1 Introduction

Hemorrhagic cystitis is a serious side effect of anticancer drugs, such as cyclophosphamide (CYP). It damages the bladder mucosal barrier, causing inflammation, excessive reactive oxygen species (ROS) production, and fibrosis, which profoundly compromise patient quality of life.^[^ ^1^ ^]^ The current clinical management primarily employs intravesical drug instillation to deliver anti‐inflammatory agents directly into the bladder lumen. Quercetin, a natural flavonoid, has emerged as a promising therapeutic candidate owing to its dual capacity for ROS scavenging and immunomodulation through macrophage phenotype switching from pro‐inflammatory M1 to anti‐inflammatory M2 polarization.^[^ ^2^ ^]^ Both pure quercetin and combination formulations containing quercetin have shown clinical efficacy in treating interstitial cystitis/bladder pain syndrome.^[^ ^3^, ^4^ ^]^ However, this treatment modality faces significant pharmacological challenges.^[^ ^5^ ^]^ Urine production and frequent voiding cycles rapidly dilute and remove the drug, thereby reducing its efficacy.^[^ ^6^ ^]^ In addition, many anti‐inflammatory drugs, including quercetin, dissolve poorly in water, break down easily in the body, and are quickly eliminated.^[^ ^7^ ^]^ These challenges highlight the critical need for improved drug delivery systems with improved solubility, stability, and retention in the bladder for long‐term treatment.

Hydrogels, which are 3D hydrophilic polymer networks capable of absorbing large amounts of water, have emerged as promising candidates for intravesical drug delivery.^[^ ^8^ ^]^ Their tunable physicochemical properties, biocompatibility, and ability to encapsulate both hydrophilic and hydrophobic drugs make them ideal candidates for controlled drug release.^[^ ^9^ ^]^ When applied to the bladder wall, hydrogels form a protective barrier that isolates the tissue from urinary irritants while gradually releasing the encapsulated therapeutics.^[^ ^10^ ^]^ Moreover, hydrogels can be engineered to respond to physiological stimuli (e.g., pH, temperature, or enzymes), allowing on‐demand drug release and degradation.^[^ ^11^ ^]^ However, several critical challenges currently limit their effectiveness in bladder cancer treatment. The initial burst‐release phenomenon often leads to transiently elevated drug concentrations, followed by subtherapeutic levels, which compromise treatment efficacy.^[^ ^7^ ^]^ Furthermore, most hydrogel‐based bioadhesives demonstrate inadequate mucoadhesive performance in the bladder environment, primarily because of weak interfacial interactions with the mucosa and disruption by continuous urine flow.^[^ ^12^ ^]^ Another significant limitation is the difficulty in achieving optimal degradation kinetics; excessively rapid degradation causes premature termination of drug release, whereas overly prolonged persistence may result in urinary retention complications.^[^ ^7^ ^]^ These limitations hinder the development of a reliable hydrogel adhesive that simultaneously achieves optimal degradation kinetics and sustains therapeutic efficacy for bladder cancer treatment.

β‐Cyclodextrin exhibits excellent biocompatibility and possesses a unique cyclic structure that enables effectively encapsulation and protection of hydrophobic drugs.^[^ ^13^ ^]^ Moreover, β‐cyclodextrin can form host–guest supramolecular interactions with aromatic residues in bioactive gelatin, making it particularly suitable for preparing dynamic hydrogels.^[^ ^14^ ^]^ These hydrogels undergo degradation through the reversible dissociation of host–guest crosslinking and enzymatic cleavage of gelatin by matrix metalloproteinases.^[^ ^15^ ^]^ Consequently, the dynamic and biodegradable nature of β‐cyclodextrin/gelatin hydrogels makes them promising candidates for controlled drug delivery.^[^ ^16^ ^]^ Nonetheless, this hydrogel had low viscosity at 37 °C. Although this characteristic is beneficial for bladder infusion, it is not conducive for the formation of an adhesive layer on the bladder wall. Competitive interactions occur as both quercetin and gelatin form complexes with β‐cyclodextrin. These interactions potentially reduce the gelation kinetics and stiffness of the hydrogel, thereby impairing its ability to achieve rapid adhesion in vivo. Additionally, cyclodextrin/gelatin hydrogels lack specific chemical functional groups for tissue interactions, significantly limiting its capacity for sustained adhesion in the urinary microenvironment.^[^ ^7^ ^]^

**Scheme 1:**
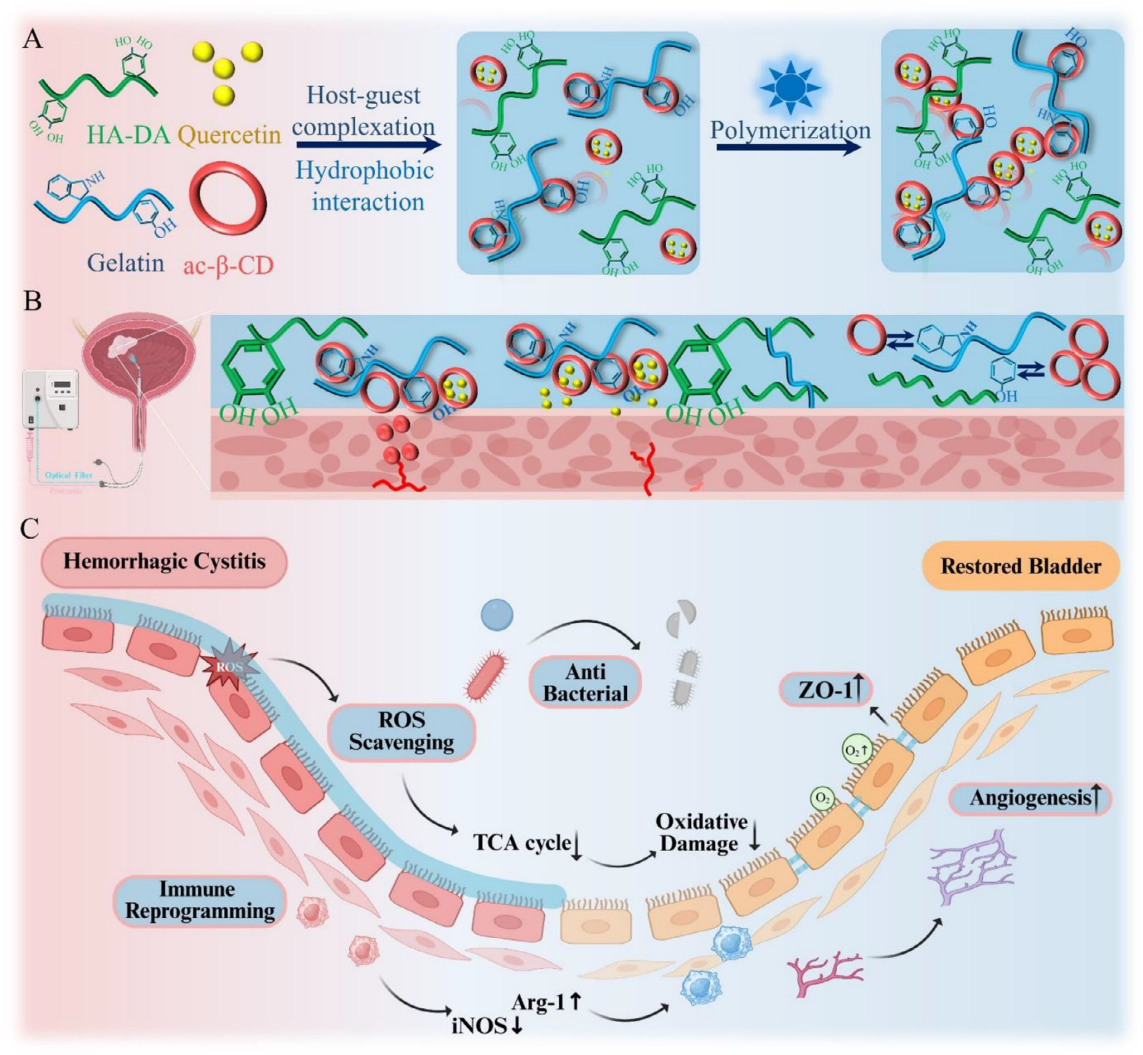
Schematic illustration of the gelatin‐based supramolecular hydrogel adhesive incorporating quercetin and dopamine‐functionalized hyaluronic acid to accelerate the healing of hemorrhagic cystitis. A) Preparation of the HGCQ hydrogel via photoinitiated polymerization following host–guest complexation. B) Illustration of the intravesical instillation and wet adhesion mechanism of the HGCQ hydrogel. C) Mechanism illustration of hemorrhagic cystitis healing facilitated by the HGCQ hydrogel, encompassing hemostasis, antibacterial, ROS scavenging, immune reprogramming and angiogenesis. (HADA, dopamine‐functionalized hyaluronic acid; HGCQ, supramolecular gelatin‐based hydrogel adhesive; ROS, reactive oxygen species; TCA, tricarboxylic acid; iNOS, inducible nitric oxide synthase; ZO‐1, zonula occludens‐1).

To address these challenges in enhancing bladder tissue healing, we propose an immunomodulatory gelatin/acrylated β‐cyclodextrin (Ac‐β‐CD) supramolecular hydrogel adhesive incorporating quercetin‐ and dopamine‐functionalized hyaluronic acid (HADA) (**Scheme** **1**, HADA/gelatin/Ac‐β‐CD/quercetin, designated as HGCQ hydrogel). Unlike conventional chemically crosslinked gelatin hydrogels, our dynamic hydrogel framework was constructed polymerization of host–guest complexes between gelatin and Ac‐β‐CD (Scheme 1A). Ac‐β‐CD also enhances the solubility of hydrophobic quercetin, establishing a primary sustained‐release system. The dopamine moieties in HADA provide multiple binding modalities through their catechol groups, enabling hydrogen bonding and π–π interaction, electrostatic interactions with tissue surfaces (Scheme 1B).^[^ ^17^ ^]^ These interactions confer reliable tissue adhesion and antioxidant properties to the HGCQ hydrogels.^[^ ^18^ ^]^ Meanwhile, the hydrophobic phenolic ring structure of dopamine and β‐cyclodextrin, combined with negatively charged hyaluronic acid (HA) and positively charged gelatin, create amounts of hydrophobic and electrostatic interactions within the hydrogel network. This sophisticated interaction network endowed the hydrogel with optimal injectability, controlled gelation kinetics, and sustained release of quercetin, all of which are critical for effective bladder therapy.

In this study, we systematically demonstrated the multifunctionality of the hydrogel, including its tissue adhesion, hemostatic performance, antibacterial properties, and anti‐inflammatory and antioxidant effects. Owing to the multiple interactions between the hydrogel and tissue surface, the HGCQ hydrogel withstood a burst pressure of up to 18.8 kPa on wet bladder tissue. Its rapid gelation and adhesion properties enabled effective hemostasis within 15 s in a rat model of liver injury. In vitro assessment confirmed the excellent cytocompatibility and potent antibacterial activity. The combined effects of the dopamine groups and quercetin significantly released ROS, thereby protecting cell viability and promoting macrophage polarization toward the M2 phenotype. RNA sequencing analysis identified the key anti‐inflammatory pathways modulated by the HGCQ hydrogel. Furthermore, we established a rat model of hemorrhagic cystitis to study the therapeutic effects of the in situ hydrogel on tissue repair (Scheme 1C). The HGCQ hydrogel effectively promoted epithelialization and tissue healing through rapid hemostasis, efficient antibacterial activity, ROS scavenging, immune reprogramming, and angiogenesis. This multifunctional hydrogel adhesive represents a promising therapeutic strategy for effective management of hemorrhagic cystitis.

## 2 Results

### 2.1 Synthesis of Supramolecular HGCQ Hydrogels

Supramolecular HGCQ hydrogel adhesives were developed by incorporating HADA and quercetin into photopolymerized host–guest gelatin/Ac‐β‐CD hydrogels to enhance intraluminal retention, wet adhesion, and the anti‐inflammatory, antibacterial, and biodegradable properties for treating hemorrhagic cystitis. Ac‐β‐CD was synthesized via β‐CD acrylation. ^1^H nuclear magnetic resonance (^1^H‐NMR) characterization confirmed the successful modification, demonstrating an average substitution of three acrylate group per β‐CD molecule (Figure S1, Supporting Information). HADA was prepared via carbodiimide‐mediated conjugation of dopamine to HA (Figure S2A, Supporting Information). Fourier transform infrared (FTIR) analysis confirmed amide bond formation, as evidenced by a characteristic peak at 1563 cm^−1^ corresponding to the N─H bending vibration (Figure S2B, Supporting Information). The conjugation was further verified and quantified by ^1^H‐NMR spectroscopy, where the emergence of new aromatic proton signals in the range of 6.6–7.2 ppm confirmed the presence of dopamine (Figure S2C, Supporting Information). The degree of dopamine grafting was calculated to be 38.2%, which is critical for the subsequent gelation behavior and tissue adhesive performance of the HGCQ hydrogel.

**Figure 1:**
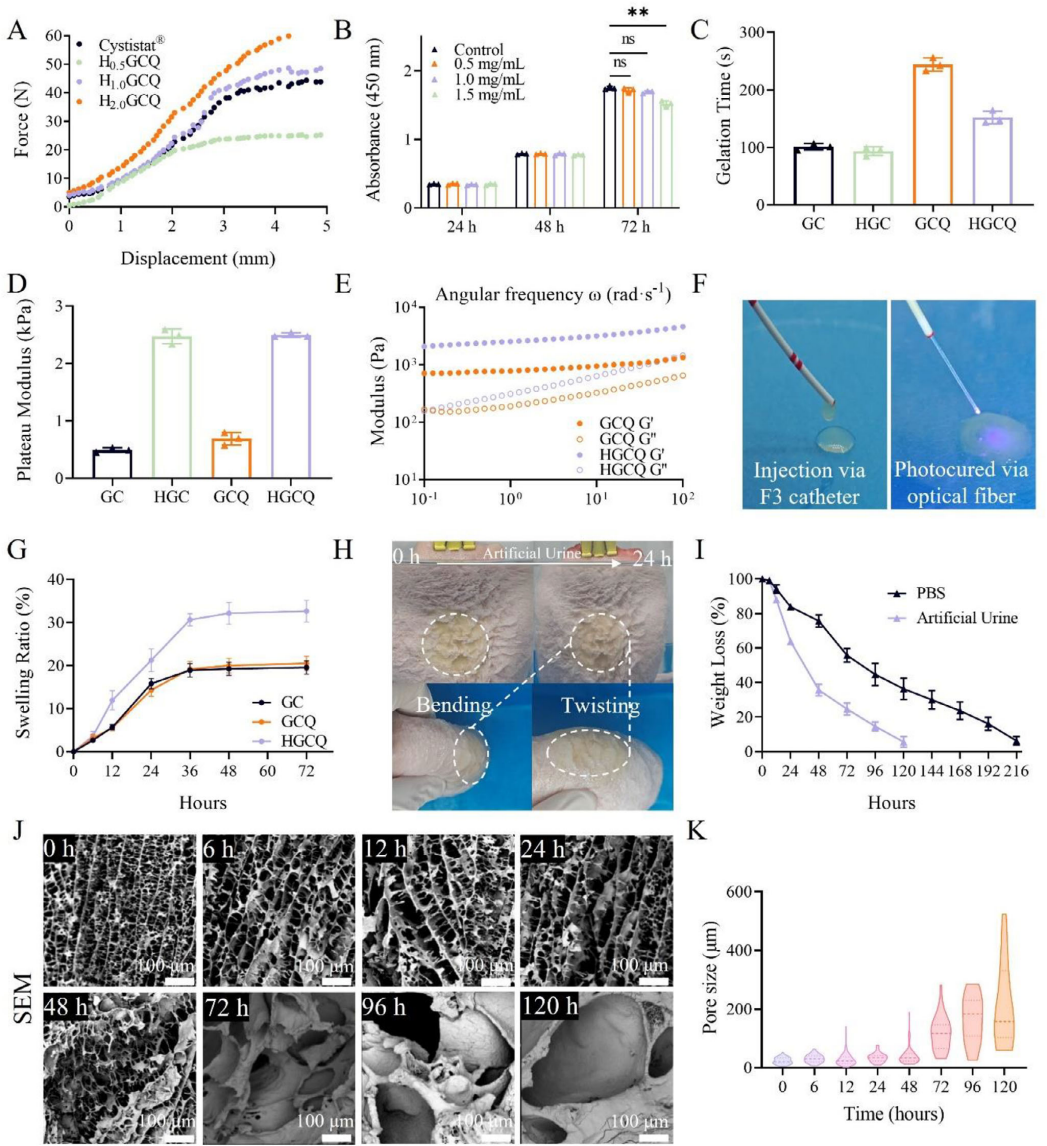
Characterization of HGCQ hydrogels. A) Average injection force of the HGCQ precursor. B) CCK8 assay for SV‐HUC‐1 cell growth of each concentration at 24, 48, and 72 h. C) Gelation time, D) plateau modulus, and E) oscillatory frequency sweep for GC, GCQ, HGC, and HGCQ hydrogels. F) Characterization of injectability and photopolymerization processes using optical fiber. G) Swelling performance of GC, GCQ, and HGCQ hydrogels in artificial urine. H) Adhesion of HGCQ hydrogel to the bladder surface in artificial urine. I) Degradation of HGCQ hydrogel in phosphate‐buffered saline (PBS) and artificial urine. J) Scanning electron microscopy (SEM) images of HGCQ hydrogel degradation in artificial urine and K) pore size analysis at corresponding time points. **p < 0.01, ns: not significant, *n* = 3. (GC, gelatin‐CD hydrogel; GCQ, gelatin‐CD‐quercetin hydrogel; HGCQ, supramolecular gelatin‐based hydrogel adhesive).

The incorporation of HADA significantly increased the viscosity of the hydrogel precursors, which improved bladder wall adhesion while simultaneously presenting challenges for microcatheter delivery. To optimize the HADA concentration, we evaluated formulations containing 0.5%, 1.0%, and 1.5% (w/v) HADA while maintaining gelatin (8%) and Ac‐β‐CD (10%) concentrations based on previous studies.^[^ ^19^ ^]^ The injectability of these precursor solutions was assessed using a 1 mL syringe fitted with a 26 G needle under compressive mechanical testing, with the clinical standard Cystistat as control. As shown in **Figure** **1**A, the required injection force increased in a concentration‐dependent manner. The 1.0% HADA formulation demonstrated an extrusion force of 45.83 N, comparable to that of Cystistat. Although the 0.5% concentration showed superior injectability, its lower catechol content compromised the wet adhesive strength. Consequently, we selected 1% HADA for the preparation of the supramolecular HGCQ hydrogel adhesive to achieve an optimal balance between microcatheter injectability and tissue adhesion.

Furthermore, to determine the optimal quercetin loading concentration while minimizing cytotoxicity, we evaluated HGCQ hydrogels loaded with 0.5, 1.0, and 1.5 mg mL^−1^ of quercetin in a 72 h Transwell co‐culture with simian virus 40‐immortalized human urothelial cell‐1 (SV‐HUC‐1). Quantitative analysis using the Cell Counting Kit 8 (CCK‐8) assay demonstrated that hydrogels incorporating 0.5 and 1.0 mg mL^−1^ quercetin supported cell proliferation and maintained high cell viability (>95%) compared to the control group at all time points (Figure 1B; Figure S3, Supporting Information). In contrast, the formulation with 1.5 mg mL^−1^ quercetin exhibited significant cytotoxicity, as indicated by a marked reduction in both absorbance values and cell viability (85.8%) at the 72 h time point. Therefore, we incorporated 1 mg mL^−1^ quercetin into the optimized HGCQ hydrogel formulation, which consisted of 1% HADA, 8% gelatin, 10% Ac‐β‐CD, and 1 mg mL^−1^ quercetin. For comparative evaluation, we prepared two control formulations: the base hydrogel (GC) containing only gelatin and Ac‐β‐CD, and an intermediate formulation (GCQ) comprising gelatin, Ac‐β‐CD, and quercetin without HADA.

### 2.2 Gelation and Dynamic Viscoelasticity of HGCQ Hydrogels

The gelation kinetics and viscoelastic properties of the HGCQ hydrogels, which are critical determinants of bladder perfusion efficiency and tissue adhesion, were differentially modulated by the incorporation of HADA and quercetin. Rheological analysis demonstrated that quercetin significantly extended the gelation time from 121.2 ± 8.6 s in GC hydrogels to 244.2 ± 11.4 s in the GCQ hydrogels (Figure 1C; Figure S4, Supporting Information). This effect is attributable to competitive host–guest complexation between quercetin and Ac‐β‐CD cavities, which hindered gelatin–sidechain interactions. Conversely, HADA incorporation in GC hydrogels (denoted as HGC) significantly accelerated gelation time to 93.5 ± 11.4 s, suggesting its function as a gelation promoter. To gain insight into the dynamic intermolecular interactions driving HGCQ hydrogel formation, we performed isothermal titration calorimetry (ITC). Upon titrating Ac‐β‐CD into gelatin and gelatin/HADA solutions, a positive peak emerged immediately within 10 s, signaling rapid dynamic binding between Ac‐β‐CD and gelatin (Figure S5, Supporting Information). Notably, the presence of HADA markedly enhanced this interaction, as evidenced by the rise in reaction heat from 0.22 to 0.55 µJ s^−1^. These results confirm that HADA reinforces the dynamic association between the Ac‐β‐CD cavity and hydrophobic motifs in gelatin, a key factor enabling rapid hydrogel formation.

Attributed to the HADA's gelation‐promoting effects through noncovalent interactions, the HGCQ hydrogel overcame the retardation induced by quercetin, yielding a faster gelation time of 152.1 ± 11.1 s compared to GCQ (Figure 1C). These accelerated gelation kinetics could facilitate achieving prompt in situ adhesion during in vivo applications. The HADA incorporation substantially enhanced the storage modulus from ≈0.5 ± 0.04 kPa for the GC hydrogel to 2.5 ± 0.03 kPa for HGCQ, whereas quercetin at 1 mg mL^−1^ loading showed no significant effect mechanical effect (Figure 1D; Figure S4, Supporting Information). Frequency sweep analyses demonstrated pronounced frequency‐dependent loss modulus behavior in both GCQ and HGCQ hydrogels, which reflects their dynamic network characteristics (Figure 1E). HADA enhanced the frequency dependence of the loss modulus, suggesting the improved viscous properties of the HGCQ hydrogel. Therefore, HADA accelerated gelation and optimized the viscoelastic balance of the HGCQ hydrogel, thus facilitating both catheter‐assisted instillation and optical fiber‐activated gelation while ensuring tissue adhesion (Figure 1F).

### 2.3 Swelling and Degradation Behaviors of HGCQ Hydrogel in Urine

Bladder's urine storage presents unique challenges for hydrogel materials.^[^ ^7^ ^]^ Urine exposure can induce hydrogel swelling, potentially compromising tissue adhesion. Moreover, it is crucial for hydrogels to maintain their urinary tract patency during degradation. Hence, we evaluated the swelling and degradation behaviors of the HGCQ hydrogel in artificial urine by simulating physiological conditions. As illustrated in Figure 1G, both GC and GCQ hydrogels showed comparable swelling profiles, reaching equilibrium at 19.3% ±1.45% after 48 h. By contrast, the HGCQ hydrogel exhibited significantly greater swelling (32.1% ± 2.29%), attributable to the high hydrophilicity and continuous erosion of HADA affecting the network's antiswelling properties (Figure S6, Supporting Information). Remarkably, the swollen HGCQ hydrogel maintained excellent adhesive stability in porcine bladder tissue during 24 h urine immersion (Figure 1H; Figure S7, Supporting Information). The HGCQ hydrogel displayed progressive degradation in artificial urine, with complete degradation occurring in 120 h, which was significantly faster than in phosphate‐buffered saline (PBS) (Figure 1I). This accelerated degradation results from the acidic pH of urine, which disrupts the dynamic bonds of the hydrogel.^[^ ^9^ ^]^ Importantly, the degradation process showed no visible fragmentation or bulk detachment, eliminating the risk of ureteral obstruction. Such a progressive degradation profile provides a sufficient therapeutic duration of 72–120 h, while preventing long‐term foreign body retention.

Scanning Electron Microscopy (SEM) analysis was performed to visually confirm the microstructural evolution during swelling and degradation. As shown in Figure 1J, the original hydrogel exhibited a well‐defined, honeycomb‐like porous structure. In contrast, after prolonged exposure to artificial urine, the hydrogel showed significant pore expansion and the emergence of structural fractures. Quantitative image analysis corroborates these observations, revealing that the average pore size increased substantially from 22.74 ± 10.90 µm (0 h) to 194.23 ± 134.01 µm after 120 h of immersion (Figure 1K). Collectively, these results indicate that the HGCQ hydrogel exhibits a time‐dependent gradient degradation behavior in artificial urine, which is crucial for preserving urinary tract patency during its degradation in bladder applications.

### 2.4 Wet Adhesion Performance of HGCQ Hydrogel on Bladder Tissues

**Figure 2:**
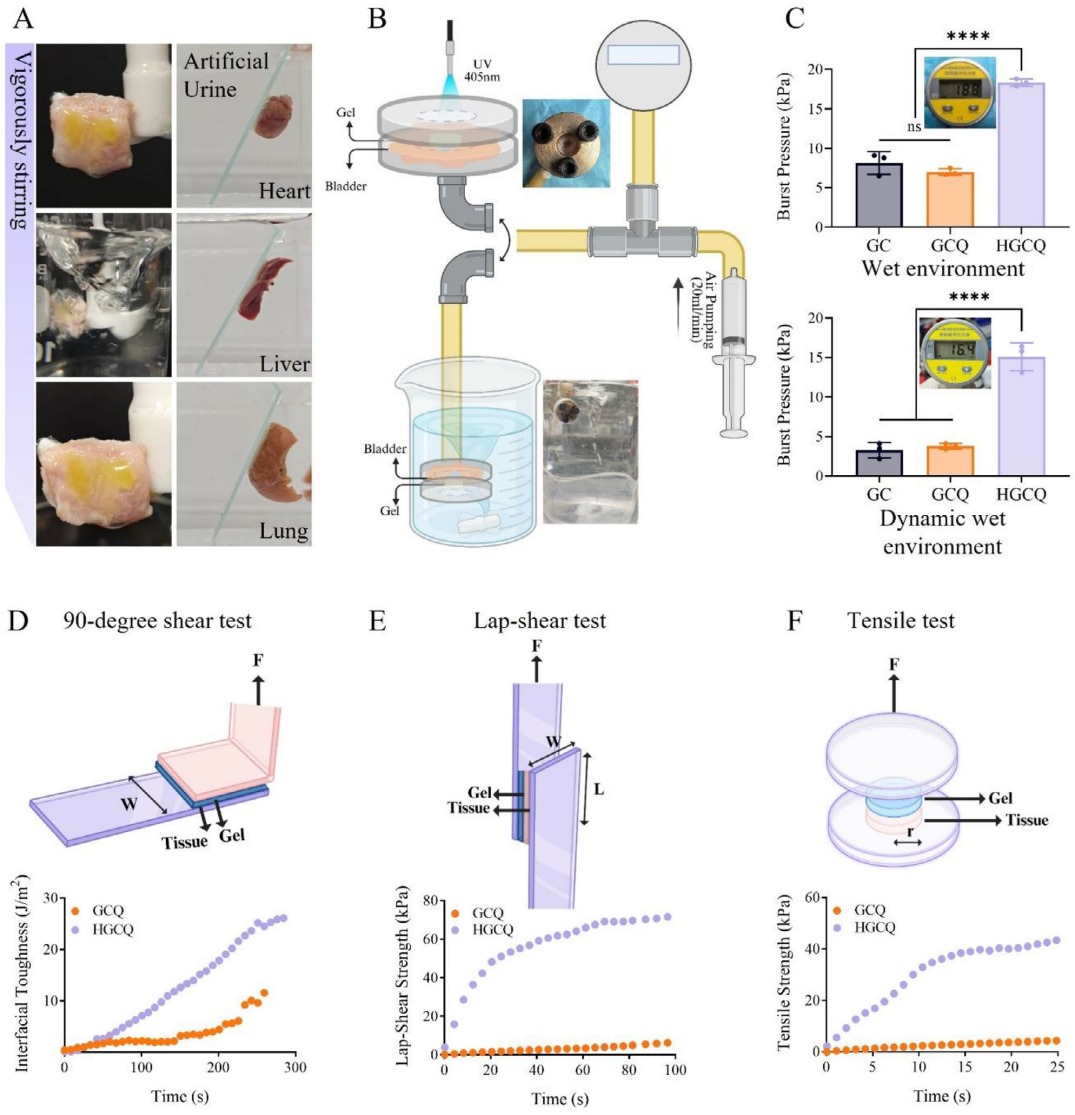
Multimodal evaluation of the wet adhesive properties of the HGCQ hydrogel. A) Wet adhesion measurement of HGCQ hydrogel under high‐shear tests and on various ex vivo rat tissues. B) Schematic of the burst pressure measurement setup under static and dynamic conditions. C) Quantitative analysis of burst pressure sealing capacity of HGCQ hydrogel on porcine bladder under both static and dynamic wet environments (****p < 0.0001, *n* = 3). D–F) Illustrations and representative curves of the D) 90° shear test, E) lap‐shear test, and F) tensile test (*n* = 3).

Hydrogel‐based bladder adhesives require robust adhesion strength to withstand stress during the bladder filling and emptying cycles.^[^ ^12^ ^]^ To assess the adhesion stability and strength of the HGCQ hydrogel to wet bladder tissue, a high‐shear test was conducted to simulate a dynamic bladder environment. As shown in **Figure** **2**A, the pig bladder tissue was fixed to the stirring head, and 200 µL of the precursor solution was applied to the tissue, followed by polymerization and immersion in artificial urine. The mixture was continuously stirred at 300 rpm for 60 min. The HGCQ hydrogel maintained a stable adhesion performance in this high‐shear dynamic environment, effectively adhering to the wet surfaces of the bladder tissue. This reliable tissue adhesion makes the hydrogel suitable for application in various tissues and organs, including the liver, lungs, and heart. To further assess the adhesive forces of the HGCQ hydrogel on bladder tissue, burst pressure was tested by applying pressure using a syringe at a rate of 20 mL min^−1^ (Figure 2B). The HGCQ hydrogel demonstrated superior pressure resistance, with a burst pressure of 18.8 kPa, compared to 7.5 kPa for the GC hydrogel and 6.7 kPa for the GCQ hydrogels. To create a more physiologically relevant simulation of the dynamic fluidic conditions within the bladder, the burst pressure test was conducted under aggressive stirring at 700 rpm (Figure 2B). Remarkably, the HGCQ hydrogel retained a high burst pressure of 16.4 kPa even in this harsh environment, demonstrating performance that was still significantly greater than the control groups (Figure 2C). This enhanced wet adhesion performance can be attributed to the viscous attachment of the hydrogel precursor, rapid in situ gelation, and interfacial bonding between the dopamine groups and the tissue.^[^ ^20^ ^]^

To systematically evaluate the adhesive force of the HGCQ hydrogels, we conducted a series of comprehensive mechanical tests, including 90° peel tests, lap‐shear, and tensile tests, using the GCQ group as a control. The 90° peel test showed that the interface toughness of the HGCQ hydrogel was 23.7 J m^−2^, nearly double that of GCQ's the 12.2 J m^−2^, which suggests enhanced resistance to delamination under peeling forces (Figure 2D). The lap‐shear test revealed that the shear strength of the HGCQ gel 71.6 kPa, considerably exceeding that of 6.2 kPa observed for the GCQ (Figure 2E). This enhancement is critical for withstanding bladder wall movements during the filling/voiding cycles. In the tensile test, the adhesion tensile strength of the HGCQ gel was determined to be 43.4 kPa (Figure 2F), which is substantially greater than the 4.3 kPa of the GCQ, demonstrating remarkable cohesion under direct pulling forces. These consistent improvements across multiple mechanical assessments highlight HGCQ's potential for reliable bladder tissue adhesion under dynamic physiological conditions.

### 2.5 Rapid Hemostatic Property and Hemocompatibility of HGCQ Hydrogel

**Figure 3:**
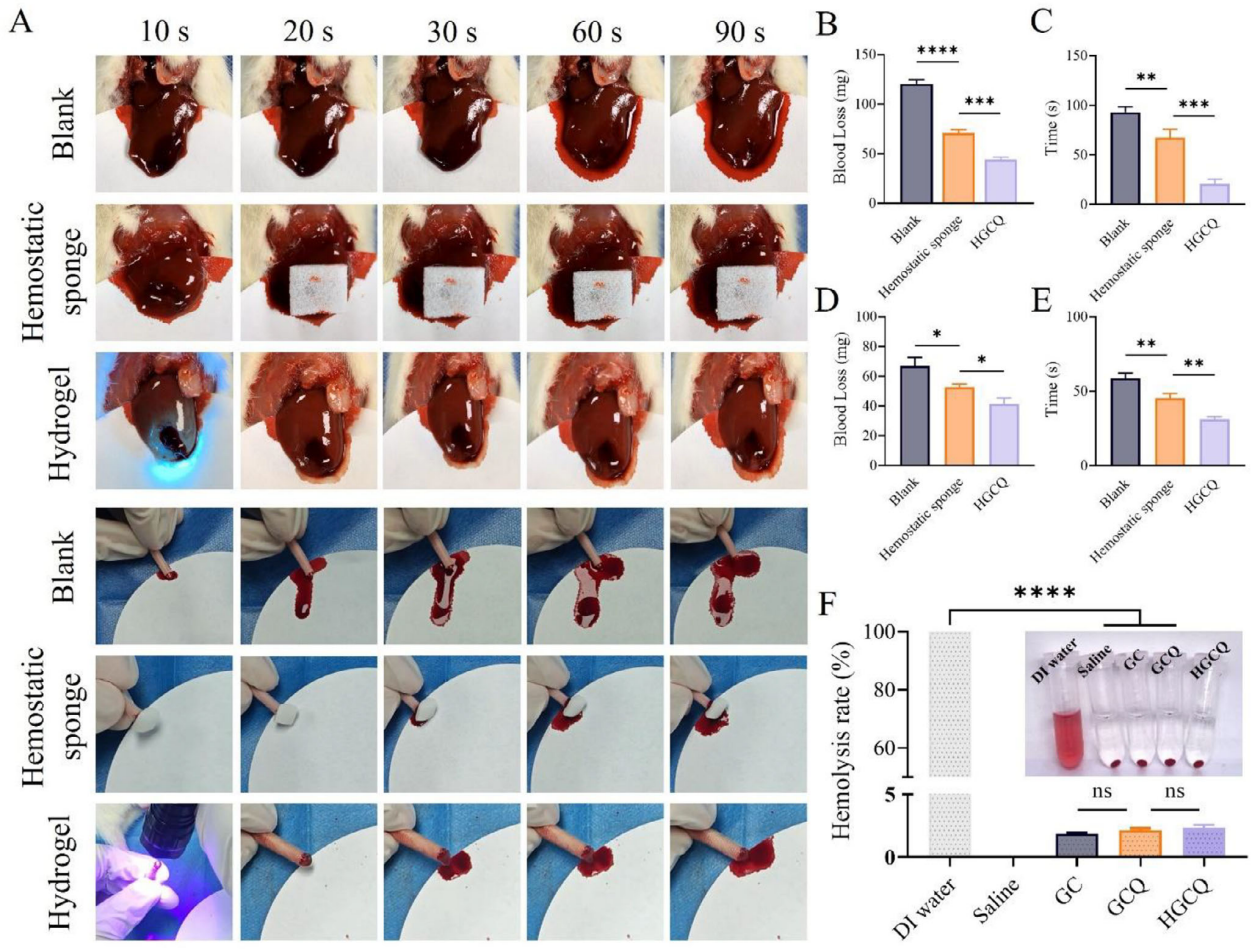
In vivo hemostatic efficacy of HGCQ in rat models. A) Representative images of the liver crucial incision bleeding model and the tail‐cut model treated with HGCQ, with blank and hemostatic sponge as controls. B–E) Quantitative statistics of blood loss and hemostasis time were performed in B,C) the liver model and D,E) the tail‐cut model, respectively. F) Hemolysis ratios of GC, GCQ, HGCQ. Inset: Photograph of hemolysis test. *p < 0.05, **p < 0.01, ***p < 0.001, ****p < 0.0001, ns: not significant, *n* = 3. (GC, gelatin‐CD hydrogel; GCQ, gelatin‐CD‐quercetin hydrogel; and HGCQ, supramolecular gelatin‐based hydrogel adhesive).

Hemorrhagic cystitis is clinically characterized by persistent bladder wall bleeding that requires immediate hemostatic management.^[^ ^21^ ^]^ The HGCQ hydrogel exhibited excellent wet tissue adhesion properties, enabling rapid hemostatic action. To evaluate the hemostatic efficacy of the HGCQ hydrogel, we employed three distinct hemorrhage models that represent progressively greater hemostatic challenges: parenchymal organ bleeding (liver), high‐flow venous injury (femoral vein), and complete transection (tail amputation). In the standardized rat liver bleeding model, a 10 mm longitudinal incision was made. Both an untreated blank group and a clinical gelatin sponge were included for comparison. As shown in **Figure** **3**A, the HGCQ hydrogel exhibited significantly enhanced hemostatic performance compared to both the gelatin hemostatic sponge and the untreated bleeding groups. The blank bleeding group showed prolonged bleeding for of 93.0 ± 4.1 s with a substantial blood loss of 120.3 ± 3.1 mg, highlighting the clinical need for effective hemostatic materials (Figure 3B,C). The gelatin sponge group reduced bleeding time to 67.3 ± 8.5 s and blood loss to 70.7 ± 3.1 mg. Remarkably, the HGCQ group achieved complete bleeding control within 21 ± 3.74 s, while limiting blood loss to 44.0 ± 2.5 mg. This performance advantage was maintained in the more challenging tail amputation model, where the HGCQ hydrogel again showed the lowest blood loss (41.33 ± 4.51 mg) and the shortest bleeding time (31.00 ± 2.00 s) (Figure 3D,E). Most notably, in the high‐flow femoral vein injury model, which best simulates pulsatile bleeding, the HGCQ hydrogel proved exceptionally effective. It curtailed the bleeding time to 34.67 ± 6.11 s and limited blood loss to 40.33 ± 3.06 mg, significantly outperforming the gelatin sponge (60.67 ± 6.11 s and 78.33 ± 3.06 mg, respectively) (Figure S9, Supporting Information). The superior hemostatic performance of the HGCQ hydrogel was mainly attributable to its rapid gelation ability, excellent wet adhesion, and moisture‐absorbing properties. These characteristics enable the HGCQ hydrogel to quickly adapt to the bleeding environment and form a stable hemostatic barrier for bleeding control.

The HGCQ hydrogel exhibited excellent hemostatic properties and favorable hemocompatibility. Hemolysis of the GC, GCQ, and HGCQ hydrogels was assessed in accordance with ISO10993‐5 standards, with physiological saline and deionized water serving as negative and positive controls, respectively.^[^ ^22^ ^]^ All hydrogels showed minimal red blood cell (RBC) damage, with hemolysis rates of 1.86% ± 0.07% for GC, 2.13% ± 0.10% for GCQ, and 2.35% ± 0.17% for HGCQ (Figure 3F), all of which are significantly below the standard threshold of 5%. Thus, the HGCQ hydrogel adhesive did not induce pathological hemolysis or trigger overactivation of the clotting system.

### 2.6 Quercetin Release from HCCQ Hydrogel

**Figure 4:**
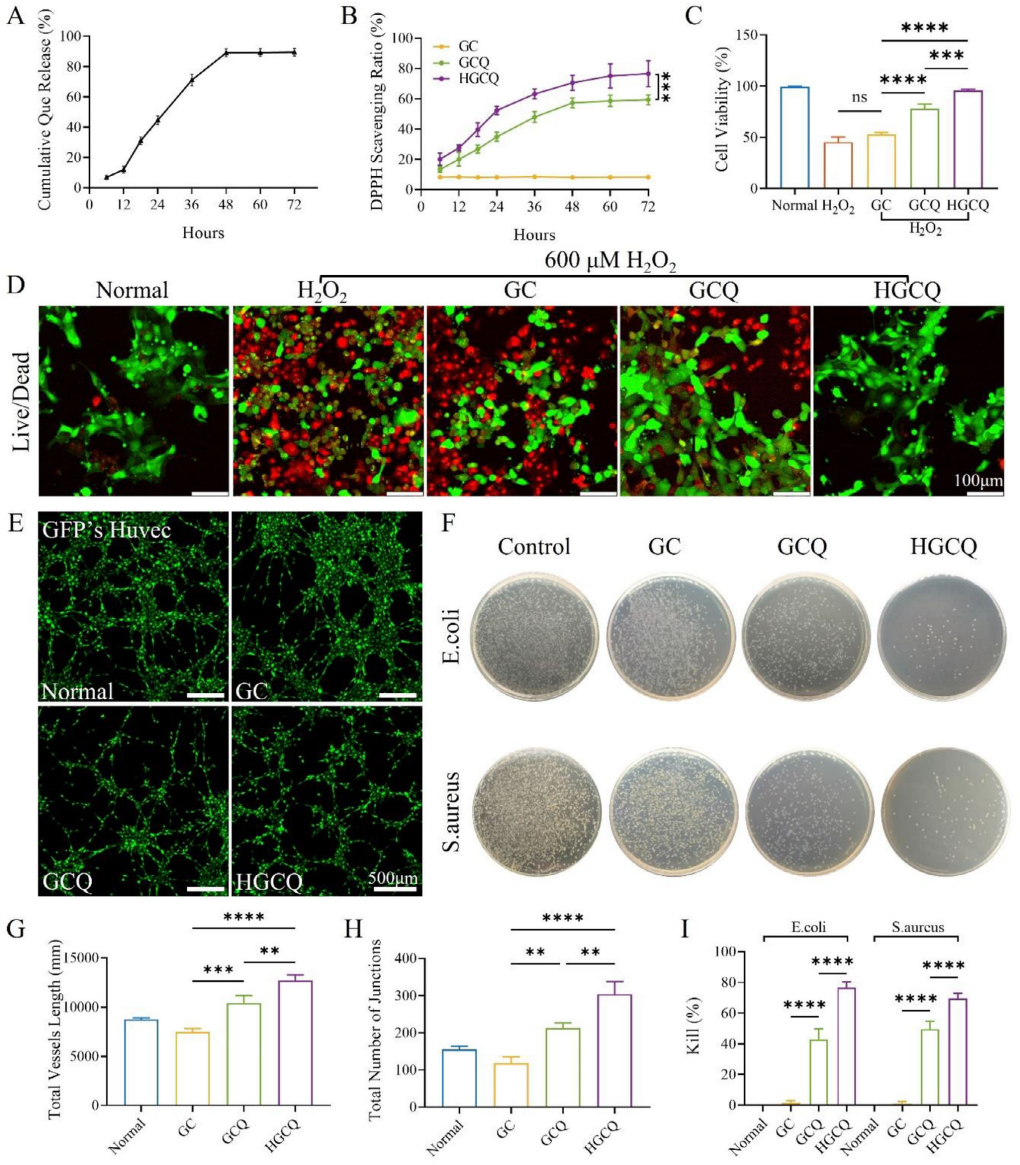
Drug release and functional characterization of HGCQ hydrogels. A) Cumulative release curve of quercetin from HGCQ hydrogels in artificial urine. B) Time‐dependent antioxidant capacity of GC, GCQ, and HGCQ hydrogels assessed by DPPH radical scavenging assay. C) Cell viability ratios and D) live/dead staining images of SV‐HUC‐1 cells co‐cultured with GC, GCQ, and HGCQ hydrogels after 24 h of incubation under 600 µm H_2_O_2_ conditions. E) Tube formation assay, F) quantitative analysis of the total junction number, and G) total vessels length of Green Fluorescent Protein (GFP)‐labeled HUVECs co‐incubated with GC, GCQ, and HGCQ hydrogels. H) Antibacterial images and I) killing ratios of GC, GCQ, and HGCQ hydrogels against *Staphylococcus aureus* and *Escherichia coli*. **p < 0.01, ***p < 0.001, and ****p < 0.0001, *n* = 3.

Anti‐inflammatory agents, such as quercetin, have a short retention time following intrabladder instillation and are rapidly eliminated via urinary excretion.^[^ ^11^ ^]^ Consequently, their long‐term anti‐inflammatory effects are limited. The hydrophobic cavity of Ac‐β‐CD in HGCQ hydrogel enables effective quercetin loading through host–guest interactions, creating a dual‐loaded system that addresses both burst release and sustained release challenges.^[^ ^23^, ^24^ ^]^ Under physiologically relevant conditions (artificial urine, 37 °C), the hydrogel demonstrated controlled release kinetics, with only 6.84% and 12.01% of quercetin released at 6 and 12 h, respectively (**Figure** **4**A; Figure S10, Supporting Information), as quantified by UV–vis spectroscopy at 385 nm. This initial phase indicates that the hydrogel effectively inhibited the initial burst release of quercetin, primarily owing to the initial dissociation of the drug from the hydrogel adhesive. Following degradation of the hydrogel, the release rates of quercetin remained consistent, as evidenced by a linear release curve that reached a plateau at 48 h. Previous studies have shown that rat models of hemorrhagic cystitis exhibit spontaneous healing within 7–10 days.^[^ ^25^ ^]^ The nearly linear drug release achieved by the HGCQ hydrogel over a 48 h duration aligns well with these therapeutic timelines.

### 2.7 Antioxidation Activity and Cytocompatibility of HCCQ Hydrogel

ROS are the major causes of inflammation during hemorrhagic cystitis.^[^ ^26^ ^]^ Overgeneration of these free radicals induces significant protein damage within tissues, creating an urgent need for efficient antioxidant interventions. Both quercetin and HADA demonstrate potent redox activities that work synergistically through a catechol–quercetin redox coupling mechanism.^[^ ^27^, ^28^ ^]^ This mechanism enables the HADA catechol groups to continuously regenerate oxidized quercetin via electron transfer, resulting in sustained ROS scavenging.^[^ ^10^ ^]^ To quantitatively evaluate antioxidant performance, we performed 2,2‐diphenyl‐1‐picrylhydrazyl (DPPH) radical scavenging assays to compare the three hydrogel formulations. The GC hydrogel showed limited antioxidant activity, achieving only 8.30% ± 0.40% DPPH scavenging after 72 h (Figure 4B). By contrast, both GCQ and HGCQ demonstrated substantially greater radical clearance capacities. HGCQ exhibited exceptional performance with 82.1% ± 1.9% scavenging efficiency, markedly exceeding the 59.3% ± 2.4% recorded for GCQ. These results highlight the superior radical‐scavenging capability of HGCQ, which is attributed to the synergistic effects of quercetin and HADA in combating oxidative stress.

The exceptional ROS‐scavenging capacity of HGCQ was demonstrated through a 600 µm H_2_O_2_ stimulation assay, highlighting its potential to counteract oxidative stress‐mediated cellular damage.^[^ ^29^ ^]^ SV‐HUC‐1 cells were cocultured with hydrogel extracts, and cell viability was assessed using a live/dead staining assay after a 24 h incubation period (Figure 4C, D). H_2_O_2_ exposure resulted in a significant reduction in cell viability (45.33% ± 4.04%), which was not significantly different from the GC group lacking active components (52.67% ± 2.52%). The GCQ hydrogel provided substantial cellular protection with 78.33% ± 3.06% viability. More impressively, HGCQ hydrogels significantly alleviated H_2_O_2_‐induced cellular injury, with cell viability improving to 97.1% ± 2.3%, demonstrating significantly enhanced efficacy compared to GCQ (*p* < 0.005). HGCQ achieved an effective removal concentration of up to 600 µm, significantly surpassing the values reported in literatures (100–300 µm).^[^ ^29^, ^30^ ^]^ This superior performance confirmed the synergistic antioxidant action of the quercetin–HADA combination in HGCQ. Additionally, primary rat bladder smooth muscle cells cultured directly on HGCQ hydrogels maintained a characteristic spindle morphology with an intact cytoskeletal architecture (Figure S11, Supporting Information), demonstrating the inherent cytocompatibility of the HGCQ hydrogel. These results confirm that the HGCQ hydrogel not only possesses excellent cell compatibility but also effectively preserves cellular viability through the dissipation of ROS.

### 2.8 Proangiogenic Activity of HGCQ Hydrogel

Hemorrhagic cystitis causes bladder wall inflammation and damages the blood vessel damage.^[^ ^11^ ^]^ This damage increases vascular permeability and bleeding, reduces the blood supply, and delays tissue repair. Therefore, proangiogenic hydrogels are crucial for therapeutic interventions.^[^ ^31^ ^]^ We assessed the angiogenic potential using an in vitro tube formation assay with human umbilical vein endothelial cells (HUVECs). HUVECs were seeded onto Matrigel‐containing hydrogel extracts. Both Matrigel alone and the GC hydrogel showed similar baseline vascular reticular structures (Figure 4E). The GCQ hydrogel significantly enhanced tube formation compared to the control. The HGCQ hydrogel with dual active components demonstrated superior proangiogenic effects.^[^ ^20^, ^22^ ^]^ Quantitative analysis revealed that the tube length and junction formation in the HGCQ group reached values of 12 710.2 ± 487.4 and 213.3 ± 10.6, respectively, which were significantly greater than those in the GCQ group (10 589.2 ± 675.7 for tube length and 303.7 ± 27.8 for junctions) (Figure 4F,G). Thus, HGCQ hydrogels possess a strong affinity for the vascular endothelium, which is critical for promoting tissue regeneration.

### 2.9 Antibacterial Activity of HCCQ Hydrogel

Bacterial infection is a major challenge in the treatment of hemorrhagic cystitis. A damaged bladder wall facilitates bacterial entry into the bladder cavity.^[^ ^32^ ^]^ Effective bacterial elimination is critical for tissue repair and prevention of complications. *Staphylococcus aureus* (Gram‐positive) and *Escherichia coli* (Gram‐negative) are key hematogeneous pathogens.^[^ ^33^ ^]^ These organisms were selected to evaluate the antibacterial effects of the hydrogels. Figure 4H shows that the HGCQ hydrogel significantly reduced the colony‐forming units (CFUs) compared to the GC and GCQ hydrogels, confirming its strong antibacterial activity in vitro against both *E. coli* and *S. aureus*. Quantitative analysis revealed a killing efficiency of >76.67% ± 3.79% for *E. coli* and 72.13% ± 3.25% for *S. aureus* (Figure 4I). The antibacterial effect of the HGCQ hydrogel resulted from sustained release combined with the contact action of HADA. Collectively, the HGCQ hydrogel displayed favorable cytocompatibility, protected cells from ROS damage through the synergistic effects of quercetin and HADA, promoted proangiogenic activity, and effectively mitigated bacterial infections, underscoring its significant potential in the treatment of hemorrhagic cystitis.

### 2.10 In Vitro Anti‐Inflammatory Activity of the HGCQ Hydrogel

**Figure 5:**
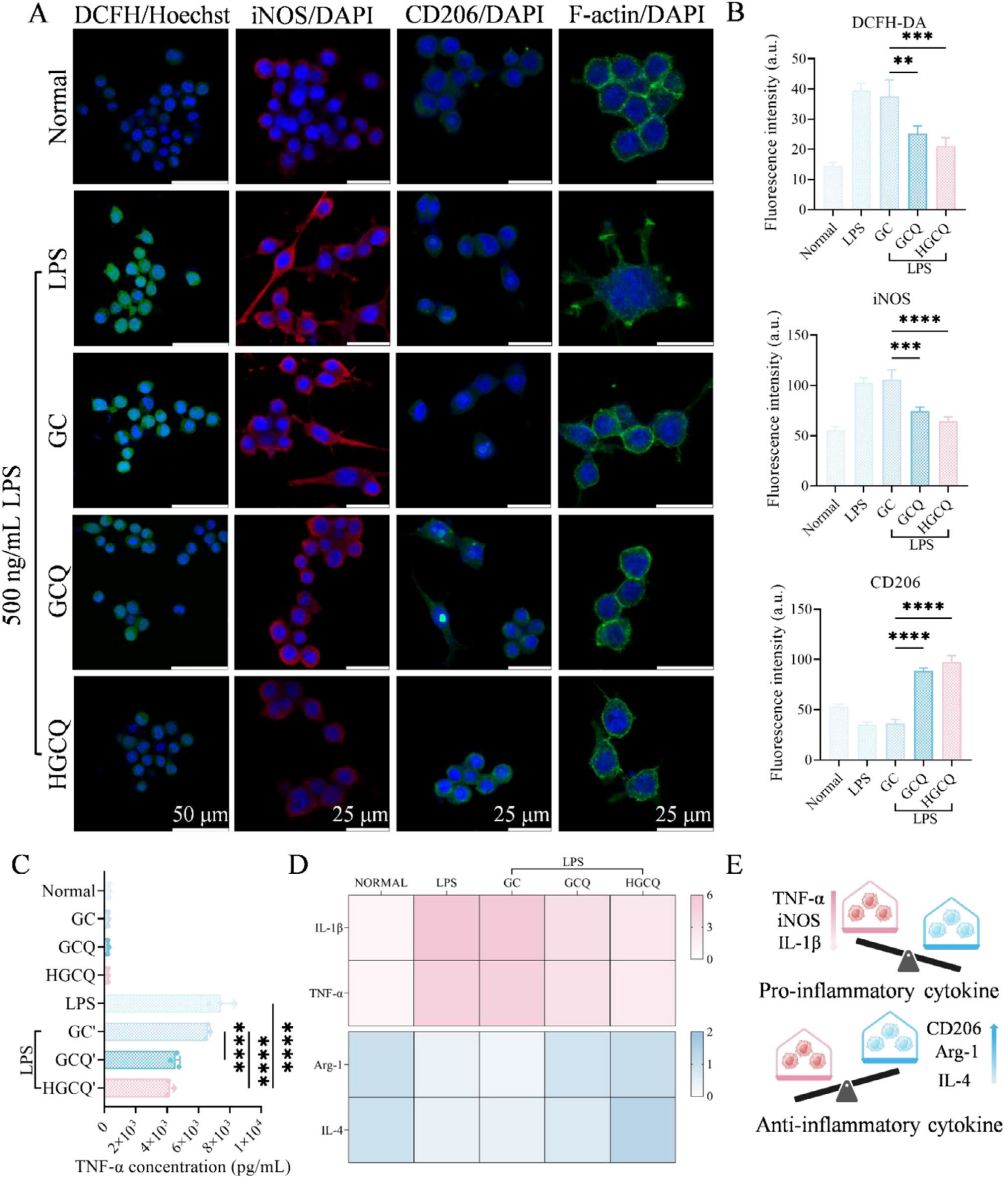
Antioxidative and immunomodulatory properties of HGCQ hydrogels in RAW264.7 macrophages. A) Immunofluorescence staining of RAW264.7 macrophages using DCFH‐DA (Hoechst 33342 for nuclei), along with iNOS (M1‐related protein), CD206 (M2‐related protein), and F‐actin staining fluorescein isothiocyanate (FITC)–phalloidin, green) to assess membrane morphology; DAPI (blue) stains nuclei. B) Fluorescence intensity analysis of DCFH‐DA, iNOS, and CD206. C) TNF‐α concentration measured via enzyme‐linked immunosorbent assay (ELISA). D) Relative messenger RNA (mRNA) expression of M1‐related genes (IL‐1β and TNF‐α) and M2‐related genes (Arg‐1 and IL‐4) in macrophages measured by reverse transcription quantitative polymerase chain reaction (RT‐qPCR). E) Illustration of inflammatory regulation by HGCQ hydrogel. **p < 0.01, ***p < 0.001, and ****p < 0.0001, *n* = 3.

Macrophages play a crucial role in the pathophysiology of hemorrhagic cystitis, primarily by generatin ROS and proinflammatory cytokines.^[^ ^34^ ^]^ To evaluate the therapeutic potential of HGCQ hydrogels, we employed RAW 264.7, an established in vitro model system for investigating anti‐inflammatory activities. We induced oxidative stress in RAW 264.7 cells using 500 ng mL^−1^ lipopolysaccharide (LPS) stimulation. The intracellular ROS levels were quantitatively assessed using a fluorescent 2',7'‐Dichlorodihydrofluorescein diacetate(DCFH‐DA) probe. Untreated control cells maintained basal ROS production, whereas LPS stimulation triggered excessive ROS accumulation (**Figure** **5**A,B). The GC hydrogels lacking antioxidant components exhibited no significant reduction in ROS production. Conversely, both the GCQ and HGCQ hydrogels demonstrated substantial ROS‐scavenging capacity, with HGCQ showing slightly higher efficacy. Thus, the HGCQ hydrogels effectively may protect cells from oxidative damage by scavenging excessive ROS.

Subsequently, the anti‐inflammatory effects of the hydrogels on RAW 264.7 macrophages were evaluated using immunofluorescence staining. Inducible nitric oxide synthase (iNOS) and CD206 served as markers for the M1 and M2 phenotypes, respectively.^[^ ^20^ ^]^ Untreated cells exhibited minimal iNOS and CD206 fluorescence, indicating an inactivated state (Figure 5A). LPS stimulation strongly increased iNOS expression, confirming the M1 phenotype. The GC hydrogel without bioactive components showed no significant changes in iNOS and CD206 levels compared with the LPS‐treated control. After treatment with the GCQ and HGCQ hydrogels for 12 h, the relative expression level of iNOS decreased markedly, whereas strong fluorescence of CD206 was observed. The similar effects observed between the GCQ and HGCQ groups suggested that both formulations effectively promoted M2 polarization (Figure 5B). Additionally, cell morphology serves as a critical indicator for assessing the macrophage activation status.^[^ ^34^ ^]^ As indicated by fluorescein isothiocyanate‐phalloidin/4′,6‐diamidino‐2‐phenylindole (DAPI) staining, LPS‐stimulated macrophages displayed a characteristic activated morphology with flattened shapes and extended pseudopodia (Figure 5A). However, HGCQ‐treated cells maintained rounded, compact morphologies, demonstrating the ability of HGCQ to modulate macrophage activation. Thus, the HGCQ hydrogel promoted M2 macrophage polarization, highlighting its therapeutic potential in inflammatory conditions.

The anti‐inflammatory efficacy of HGCQ hydrogel was further assessed by quantifying inflammatory cytokine tumor necrosis factor‐α (TNF‐α) levels using enzyme‐linked immunosorbent assay. As shown in Figure 5C, RAW 264.7 cells showed low baseline TNF‐α expression (339.45 ± 26.09 pg mL^−1^). Importantly, none of the hydrogels induced TNF‐α production when cocultured with macrophages, confirming their superior biocompatibility. Following LPS stimulation, TNF‐α level increased dramatically to 7452.8 ± 832.2 pg mL^−1^, indicating a strong inflammatory response. Although GC hydrogel showed similarly elevated TNF‐α expression levels, both GCQ and HGCQ hydrogels significantly reduced TNF‐α levels to 4535.8 ± 266.6 and 4186.9 ± 100.7 pg mL^−1^, respectively. Further analysis by reverse transcription quantitative polymerase chain reaction demonstrated that GCQ and HGCQ hydrogels downregulated proinflammatory genes (interleukin (IL)‐1β and TNF‐α) while upregulating anti‐inflammatory markers (Arg‐1 and IL‐4) compared to LPS‐stimulated controls (Figure 5D; Figure S13, Supporting Information). Consequently, the HGCQ hydrogel demonstrated good inflammatory transformation capability from the proinflammatory to anti‐inflammatory states (Figure 5E).

### 2.11 RNA Sequencing Analysis of RAW 264.7 Cells with HGCQ Coculture

**Figure 6:**
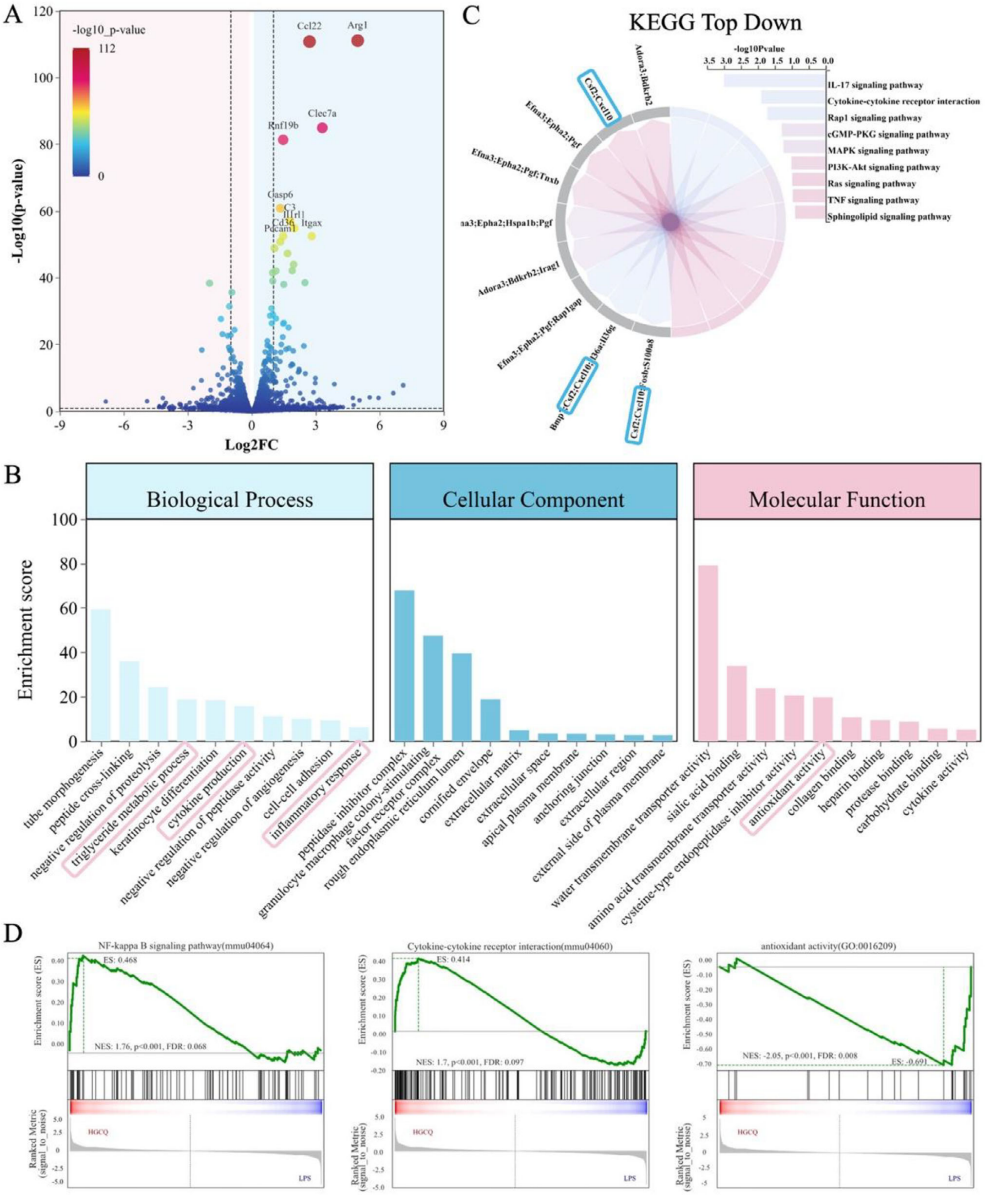
Transcriptome sequencing analysis of RAW264.7 macrophage responses to LPS stimulation with/without HGCQ treatment. A) Volcano plot depicting differentially expressed genes (DEGs) in RAW264.7 cells. B) Chord diagram illustrating the functions of downregulated KEGG‐enriched pathway. C) GO enrichment analysis of DEGs between LPS+HGCQ and LPS‐only groups. D) GSEA analysis revealed enrichment of gene sets associated with the NF‐𝜅B pathway, cytokine–cytokine receptor interactions, and antioxidant activity. (LPS, lipopolysaccharide; GO, gene ontology; KEGG, Kyoto Encyclopedia of Genes and Genomes; GSEA, gene set enrichment analysis).

To lucidate the impact of the HGCQ hydrogel on macrophage immunoregulation, RNA sequencing analysis was conducted on LPS‐treated RAW 264.7 cells incubated with HGCQ leachate for 12 h, with LPS‐treated cells as the control. Bioinformatic analysis identified 2025 differentially expressed genes (DEGs), including 113 upregulated and 90 downregulated genes, showing statistically significant differences between the LPS and HGCQ groups (Figure S14, Supporting Information). Several DEGs including Arg‐1, Ccl22, and Clec7a were associated with anti‐inflammatory regulation (**Figure** **6**A). Kyoto Encyclopedia of Genes and Genomes (KEGG) pathway analysis identified the key signaling pathways involved in anti‐inflammatory effects (Figure 6B). The most downregulated DEGs in HGCQ‐treated cells were linked to interleukin (IL‐17) signaling, cytokine–cytokine receptor interactions, and TNF signaling. A chord diagram further illustrated that the HGCQ‐mediated reduction in inflammation involved the downregulation of critical genes related to cystitis, such as Csf2 and Cxcl10.^[^ ^35^ ^]^

Gene Ontology analysis was conducted to assess the functional roles of the DEGs, including biological processes, molecular functions, and cellular components. The genes downregulated in HGCQ‐treated cells were primarily enriched for inflammatory responses, triglyceride metabolism, cytokine production, granulocyte macrophage colony stimulation, antioxidant activity, and cytokine activity (Figure 6C). Gene set enrichment analysis (GSEA) showed that HGCQ treatment significantly enriched pathways related to NF‐κB signaling, cytokine–cytokine receptor interactions, and antioxidant activity (Figure 6D). Overall, HGCQ suppressed oxidative stress and inflammation in RAW 264.7, primarily by regulating nuclear factor kappa‐B (NF‐κB) signaling, IL‐17 signaling, and oxidative stress pathways.

### 2.12 In Vivo HGCQ Hydrogel Instillation for the Repair of Hemorrhagic Cystitis

**Figure 7:**
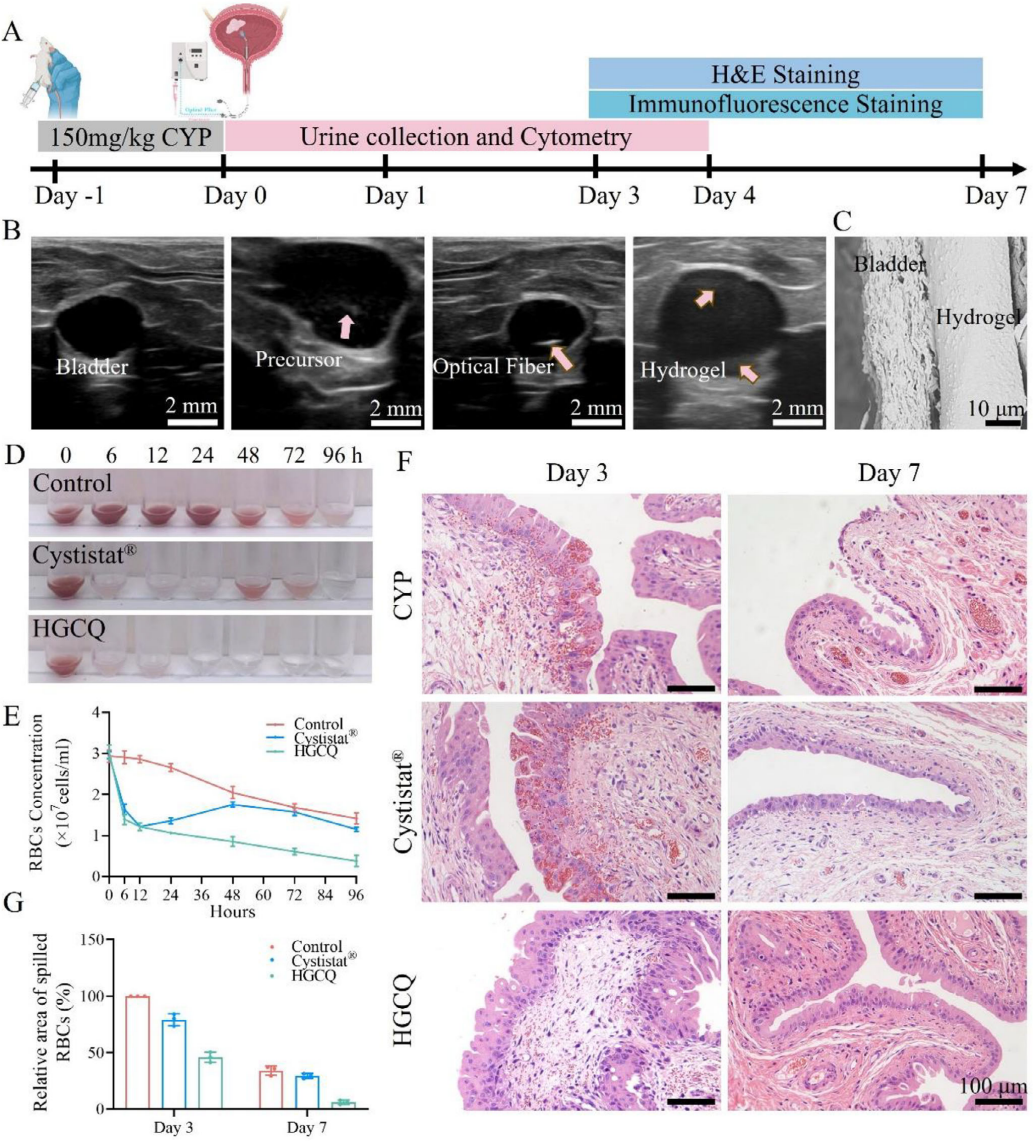
Therapeutic efficiency of HGCQ hydrogel in hemorrhagic cystitis treatment. A) Experimental timeline schematic detailing hydrogel administration and evaluation time points. B) Ultrasound images monitoring the entire process of the animal experiments. C) Scanning electron microscopy characterization of the adhesion contact surface between HGCQ hydrogel and bladder tissue. D) Urine samples showing progressive hematuria resolution and E) statistical analysis of blood cell counts. F) H&E staining of rat bladder tissues at days 3 and 7 post treatments. G) Quantitative comparison analysis of extravasated erythrocyte infiltration areas in bladder sections. (H&E, hematoxylin and eosin; CYP, cyclophosphamide; RBC, red blood cell; HGCQ, supramolecular gelatin‐based hydrogel adhesive).

Cyclophosphamide is a widely used immunosuppressive and chemotherapeutic agent in cancer treatment. However, its metabolites can cause hemorrhagic cystitis by damaging the urothelial lining, leading to inflammation, bleeding, and impaired bladder function.^[^ ^27^ ^]^ The injectable HGCQ hydrogel demonstrates extensive flowability, allowing complete coverage of the injured tissue surface upon administration. After photopolymerization, a stable adhesive layer is formed, which promotes hemostasis. Moreover, the hydrogel possesses strong antibacterial, antioxidant, and anti‐inflammatory properties, making it particularly suitable for treating hemorrhagic cystitis. To assess the therapeutic potential of the hydrogel, we constructed a rat model by intraperitoneally injecting cyclophosphamide at a dose of 150 mg kg^−1^ following our established protocol.^[^ ^21^ ^]^ The experimental procedure for the hydrogel treatment is illustrated in **Figure** **7**A. The HGCQ precursor (200 µL of HGCQ precursor was administered into the damaged bladder via a 1 mm outer diameter F3 catheter and retained for 1 min to ensure complete mucosal coverage. After draining the excess fluid, in situ polymerization was achieved using 405 nm light (10 W cm^−2^) delivered through an optical fiber for 1 min. The entire process was monitored by real‐time ultrasonography (Figure 7B). Subsequent analysis by scanning electron microscopy confirmed that the polymerized hydrogel formed a tightly adherent 23 µm thick layer on the bladder wall (Figure 7C). For experimental controls, we used equivalent volumes of the commercial agent Cystistat and included an untreated group for comparison.

Hematuria assessment serves as a primary clinical indicator reflecting both disease severity and treatment response.^[^ ^35^ ^]^ We collected rat urine samples at 6, 12, 24, 48, 72, and 96 h post‐treatment to monitor the hematic efficacy in hemorrhagic cystitis. As shown in Figure 7D, both HGCQ and Cystistat reduced hematuria within 6 h, suggesting effective protection of the bladder's glycosaminoglycan (GAG) layer. However, the Cystistat group showed hematuria recurrence at 48 h, whereas HGCQ maintained protection with no visible blood in urine after 24 h. The untreated CYP controls displayed persistent severe hematuria with detectable blood‐tinged urineat 96 h. Quantitative analysis of RBC counts confirmed these results. The HGCQ group showed a rapid RBC decline from 3.0×10⁷ to 1.4×10⁷ at 6 h, further decreasing to 0.39 ± 0.11×10⁷ by 96 h (Figure 7E). By contrast, CYP controls and Cystistat‐treated animals maintained higher RBC counts ((1.42 ± 0.11) ×10⁷ and (1.15 ± 0.06) ×10⁷ respectively) at 96 h. These results demonstrate HGCQ's superior and sustained hematuria control, resulting from its enhanced tissue adhesion and therapeutic properties.

Hematoxylin and eosin (H&E) staining was performed on the bladder during the repair phase on days 3 and 7 to assess bleeding, inflammation, and tissue healing. As shown in Figure 7F, the CYP control group showed pronounced diffuse hemorrhage and dense inflammatory cell infiltration on day 3. Although some improvement was observed on day 7, persistent erythrocyte accumulation indicated sustained bleeding and inflammation. The Cystistat group similarly exhibited substantial erythrocyte deposition at day 3, accompanied by disorganized detrusor muscle architecture. Although mucosal regeneration improved by day 7, the muscularis propria exhibited erythrocyte infiltration and structural laxity. In comparison, HGCQ‐treated bladders demonstrated minimal erythrocyte dispersion on day 3, with markedly reduced inflammation and preserved mucosal‐muscular integrity. Quantitative analysis of RBC density (relative to tissue area) confirmed HGCQ achieved the lowest levels of 45.28% ± 4.56% on day 3 and 6.17% ±1.84% on day 7, respectively (Figure 7G). Notably, in the absence of quercetin, hemorrhage in the HGC group remained significantly higher than that in the HGCQ group (Figure S15, Supporting Information), indicating that quercetin plays an essential role in enhancing the hemostatic and healing functions of the hydrogel. Thus, the HGCQ hydrogel adhesive effectively promoted tissue repair in hemorrhagic cystitis via a quercetin‐augmented HGC mechanism.

### 2.13 Mechanisms of Promoted Hemorrhagic Cystitis Healing

**Figure 8:**
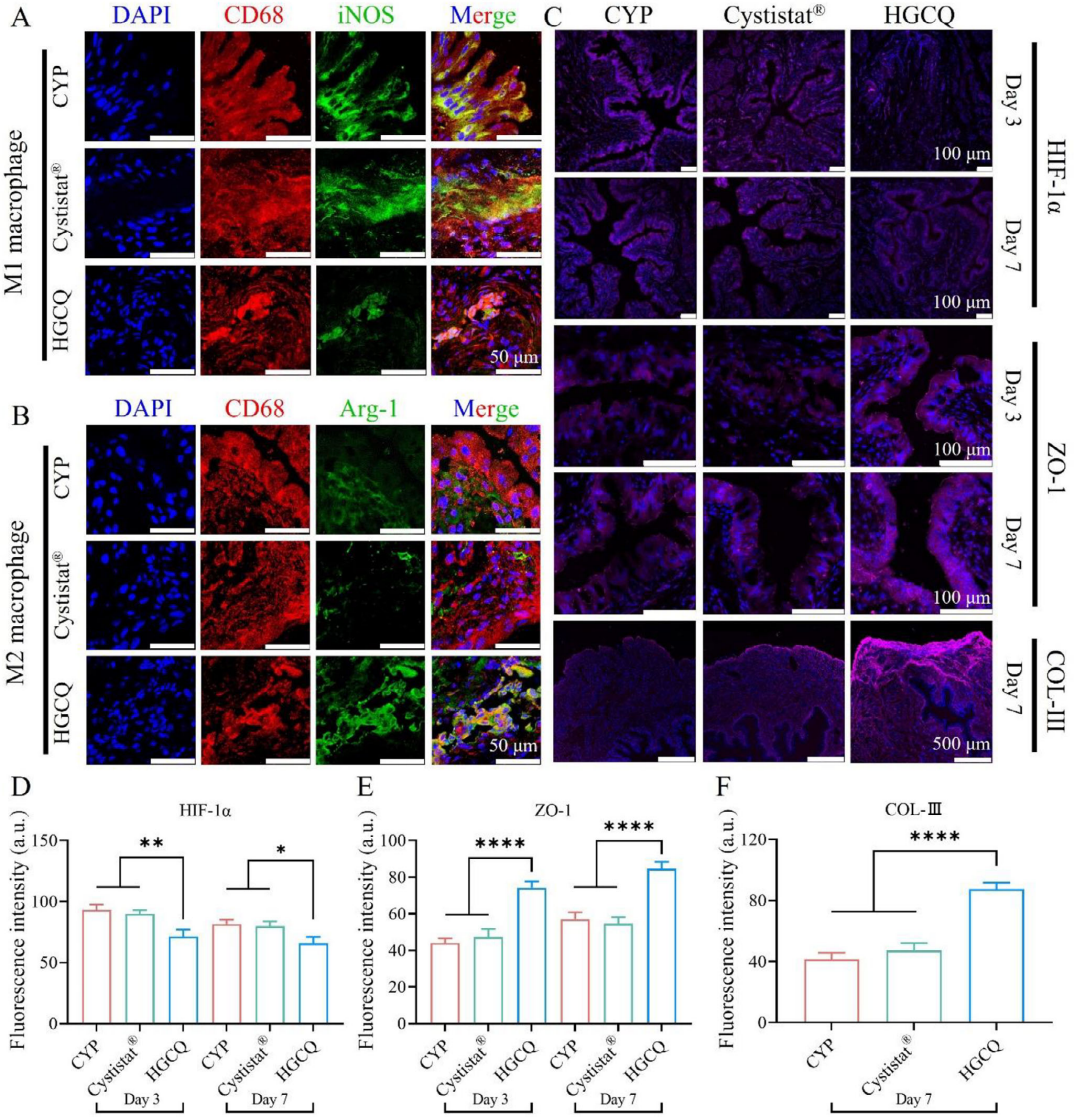
Inflammatory modulation of HGCQ hydrogel in hemorrhagic cystitis healing. A,B) Immunofluorescence images showing macrophages staining for A) M1 phenotype marker (CD68/iNOS/DAPI) and B) M2 phenotype markers (CD68/Arg1/DAPI) in the regenerated tissues on day 3. C) Staining images for HIF‐1α and ZO‐1 after 3 and 7 days, along with COL‐III staining at day 7. D–F) Quantitative statistical analysis of D) HIF‐1α, E) ZO‐1, and F) COL‐III fluorescence. *p < 0.05, **p < 0.01, and ****p < 0.0001, *n* = 3. (HGCQ, supramolecular gelatin‐based hydrogel adhesive; iNOS, inducible nitric oxide synthase; COL‐III, type III collagen; ZO‐1, zonula occludens‐1; HIF‐1α, hypoxia‐inducible factor‐1α; DAPI, 4′,6‐diamidino‐2‐phenylindole.).

To elucidate the regulatory mechanisms of the HGCQ hydrogel in the healing process of hemorrhagic cystitis, we examined macrophage polarization patterns in bladder tissues 3 days post‐treatment using immunofluorescence staining. As shown in **Figure** **8**A, the CYP control and Cystistat groups exhibited a higher abundance of M1 macrophages, as indicated by elevated levels of iNOS. The HGCQ group showed diminished iNOS fluorescence signals, indicating the presence of fewer M1 macrophages. HGCQ‐treated tissue had the most M2 macrophages (stronger Arg1 signals) compared to both the CYP and Cystistat groups (Figure 8B). Further evaluation of hypoxia‐inducible factor‐1α (HIF‐1α) expression at 3 and 7 days post‐healing revealed HGCQ treatment significantly reduced HIF‐1α levels relative to CYP, Cystistat, and HGC controls (Figure 8C,D; Figure S15B, Supporting Information). Overall, HGCQ effectively alleviated tissue hypoxia and prevented oxidative stress‐mediated damage in the bladder.

To assess urothelial barrier regeneration, we performed immunofluorescence staining for zonula occludens‐1 (ZO‐1), a critical tight junction protein essential for maintaining bladder mucosal integrity, on postoperative days 3 and 7.^[^ ^36^ ^]^ Immunofluorescence analysis revealed progressive ZO‐1 expression in all groups during recovery from hemorrhagic cystitis (Figure 8C). However, CYP‐treated bladders showed markedly reduced ZO‐1 levels (Figure 8E), indicating substantial urothelial barrier dysfunction. Although Cystistat treatment provided initial GAG layer protection, this effect was temporary, resulting in inconsistent and suboptimal ZO‐1 expression on day 7. By contrast, the HGCQ hydrogel treatment induced significant ZO‐1 upregulation at both time points, highlighting its superior capacity to restore urothelial barrier function through tight junction reinforcement. Moreover, HGCQ hydrogel significantly enhanced type III collagen (COL‐III) deposition compared to the CYP, Cystasis‐treated group, and HGC hydrogel group (Figure 8C,F; Figure S15C,D, Supporting Information). Collectively, these results demonstrate that the HGCQ hydrogel promoted the comprehensive recovery of hemorrhagic cystitis through multiple synergistic mechanisms, including preservation of tissue integrity, potent anti‐inflammatory and antioxidant effects, restoration of tight junction proteins, and promotion of endothelial cell medium (ECM) remodeling. The multifunctional therapeutic action of HGCQ, which combines these complementary biological effects, highlights its significant clinical potential for the effective management of hemorrhagic cystitis.

## 3 Discussion

This study successfully developed a gelatin‐based supramolecular HGCQ hydrogel adhesive, which integrates HADA and quercetin into a photopolymerized host–guest gelatin/Ac‐β‐CD network, achieving efficient treatment in the complex physiological environment of hemorrhagic cystitis. Through molecular‐level synergistic integration, HGCQ forms a multiple functional system with bioactivity to address key challenges in drug delivery and tissue repair within the bladder.

The incorporation of HADA critically enhances both gelation kinetics and wet adhesion. HGCQ gels within 152.1 s, which was significantly faster than HADA‐free formulations, ensuring rapid solidification under flow conditions.^[^ ^37^ ^]^ Moreover, the catechol‐mediated adhesion, combined with the dynamic network, allows the hydrogel to withstand repetitive bladder contractions, as evidenced by a high burst pressure of 18.8 kPa. This rapid yet adaptive adhesion is vital for retention in a wet, mechanically active environment. Furthermore, traditional hydrogels often face the dual dilemma of burst release and short release cycles in drug delivery.^[^ ^38^, ^39^ ^]^ By leveraging host–guest interactions between Ac‐β‐CD and quercetin within a dynamic gelatin network, HGCQ achieves near‐linear release kinetics over 48 h, effectively mitigating burst release and maintaining therapeutic drug levels. This behavior stems from the reversible crosslinks that act as a molecular “gatekeeper,” enabling sustained and regulated release ideal for long‐term anti‐inflammatory therapy.^[^ ^40^ ^]^ Additionally, HGCQ exhibited controllable degradation characterized by surface erosion‐like behavior in artificial urine, completing clearance within 120 h without fragmentation. This urinary‐responsive degradation aligns with tissue regeneration timelines, reducing risks associated with premature breakdown or foreign‐body persistence.

In addition to serving as a physical barrier and drug carrier, the HGCQ hydrogel actively regulates the local microenvironment and promotes tissue regeneration through the synergistic effects of its components. Quercetin and the catechol groups in HADA achieve sustained ROS scavenging capabilities through a redox coupling mechanism, effectively protecting cells from oxidative damage. This interaction also modulates the NF‐κB/IL‐17 signaling pathway at both mRNA and protein levels, promoting the polarization of macrophages from the M1 to M2 phenotype, thereby alleviating the inflammatory response. HGCQ extracts significantly enhanced the tube formation ability of HUVEC, increasing blood supply necessary for tissue repair. Additionally, it exhibits over 70% bactericidal activity against both *E. coli* and *S. aureus*, effectively preventing secondary infections. In animal models, HGCQ treatment significantly reduced hematuria, promoted the expression of ZO‐1 tight junction protein, and increased collagen type III deposition, histologically confirming its ability to effectively restore the structural and functional integrity of the bladder epithelial barrier. Overall, the HGCQ supramolecular hydrogel adhesive demonstrates multifunctional effects with synergistic enhancement, offering a promising new solution for the clinical treatment of hemorrhagic cystitis.

## 4 Conclusion

We developed a supramolecular HGCQ hydrogel adhesive by integrating HADA and quercetin into a photocrosslinked gelatin/Ac‐β‐CD network, creating a multifunctional system capable of effectively addressing the complex therapeutic challenges of hemorrhagic cystitis. HADA incorporation endowed the hydrogel with rapid gelation kinetics and robust wet tissue adhesion properties. Through Ac‐β‐CD complexation, we achieved a unique secondary sustained‐release system capable of linear quercetin release throughout the critical 48 h therapeutic window. The hydrogel exhibited smart urea‐responsive degradation, enabling spontaneous clearance after fulfilling its therapeutic function. Importantly, the HGCQ hydrogel demonstrated potent antioxidant activity through ROS scavenging and effectively modulated inflammatory responses by regulating NF‐κB and IL‐17 signaling pathways. This dual action promoted macrophage polarization toward the anti‐inflammatory M2 phenotype, creating a regenerative microenvironment. In animal models of hemorrhagic cystitis, the HGCQ adhesive formed a protective layer on the bladder mucosa that achieved rapid hemostasis while facilitating tissue transition from proinflammatory to anti‐inflammatory states. These effects enhance epithelial regeneration and collagen deposition, thereby significantly accelerating the healing of hemorrhagic cystitis. Overall, the HGCQ hydrogel adhesive has great potential for promoting the healing of hemorrhagic cystitis and other wounds.

## Conflict of Interest

The authors declare no conflict of interest.

## Supporting information



Supporting Information

## Data Availability

The data that support the findings of this study are available from the corresponding author upon reasonable request.

## References

[1] A. Mohammad , M. A. Laboulaye , C. Shenhar , A. D. Dobberfuhl , Nat. Rev. Urol. 2024, 21, 433. 38326514 10.1038/s41585-023-00850-y

[2] Y. Zhu , Z. Xiu , X. Jiang , H. Zhang , X. Li , Y. Feng , B. Li , R. Cai , C. Li , G. Tao , J. Nanobiotechnol. 2025, 23, 205. 10.1186/s12951-025-03275-4 PMC11900060 40075491

[3] T. C. Theoharides , D. Kempuraj , S. Vakali , G. R. Sant , Can. J. Urol. 2008, 15, 4410. 19046494

[4] F. Katske , D. A. Shoskes , M. Sender , R. Poliakin , K. Gagliano , J. Rajfer , Tech. Urol. 2001, 7, 44. 11272677

[5] A. Guo , Q. Cao , H. Fang , H. Tian , J. Controlled Release 2025, 385, 114021. 10.1016/j.jconrel.2025.114021 40645293

[6] K. Chen , Z. Wu , Y. Liu , Y. Yuan , C. Liu , Adv. Funct. Mater. 2021, 32, 2109687.

[7] Y. Wu , X. Gu , X. Chen , Y. Cui , W. Jiang , B. Liu , J. Mater. Chem. B 2024, 12, 2938. 38426380 10.1039/d3tb02837b

[8] J. G. Lee , J. Petraccione , K. A. Trese , A. C. Hughes , T. R. Ausec , M. Salzmann‐Sullivan , L. J. Su , M. T. Kim , S. Roh , A. P. Goodwin , F. X. Feng , T. W. Flaig , C. W. Shields , Adv. Mater. 2025, 37, 2505231. 10.1002/adma.202505231 PMC12233162 40611758

[9] Y. Li , T. Li , J. Feng , B. Liu , Z. Wang , J. He , Z. Chen , R. Tao , H. Wang , K. Fan , Y. Sun , J. Wang , B. Guo , G. Zhang , Biomaterials 2025, 321, 123320. 40209592 10.1016/j.biomaterials.2025.123320

[10] Y. Wang , Y. Zhang , Y. P. Yang , M. Y. Jin , S. Huang , Z. M. Zhuang , T. Zhang , L. L. Cao , X. Y. Lin , J. Chen , Y. Z. Du , J. Chen , W. Q. Tan , Bioact. Mater. 2024, 35, 330. 38379700 10.1016/j.bioactmat.2024.02.010 PMC10876488

[11] B. L. Wang , X. Gao , K. Men , J. Qiu , B. Yang , M. L. Gou , M. J. Huang , N. Huang , Z. Y. Qian , X. Zhao , Y. Q. Wei , Int. J. Nanomed. 2012, 7, 2239. 10.2147/IJN.S29416 PMC3357976 22661886

[12] H. An , M. Zhang , Z. Huang , Y. Xu , S. Ji , Z. Gu , P. Zhang , Y. Wen , Adv. Mater. 2024, 36, 2310164. 10.1002/adma.202310164 37925614

[13] R. Li , X. Zhang , Q. Zhang , H. Liu , J. Rong , M. Tu , R. Zeng , J. Zhao , J. Appl. Polym. Sci. 2015, 133, 43072.

[14] T. Wang , J. Chen , C. Shu , X. Shen , Y. Fu , M. Li , Z. Luo , Acta Biomater. 2025, 194, 396. 39884521 10.1016/j.actbio.2025.01.049

[15] X. Zhang , B. Yang , L. Feng , X. Xu , C. Wang , Y. W. Lee , M. Wang , X. Lu , L. Qin , S. Lin , L. Bian , G. Li , Bioact. Mater. 2024, 41, 440. 39188381 10.1016/j.bioactmat.2024.07.036 PMC11347042

[16] X. Ge , H. Wen , Y. Fei , R. Xue , Z. Cheng , Y. Li , K. Cai , L. Li , M. Li , Z. Luo , Biomaterials 2023, 299, 122184. 37276796 10.1016/j.biomaterials.2023.122184

[17] M. Fan , J. Yang , L. Zhen , J. Zhu , K. Liang , J. Li , Chem. Eng. J. 2025, 509, 161262.

[18] P. Wang , G. Lu , X. Xiao , X. Li , L. Tong , Y. Wang , Y. Cheng , J. Liang , Y. Fan , X. Zhang , Y. Sun , Small 2025, 21, 2501773. 10.1002/smll.202501773 40411892

[19] X. Xu , Q. Feng , X. Ma , Y. Deng , K. Zhang , H. S. Ooi , B. Yang , Z.‐Y. Zhang , B. Feng , L. Bian , Biomaterials 2022, 289, 121802. 36152514 10.1016/j.biomaterials.2022.121802

[20] S. Yang , Y. Zhu , C. Ji , H. Zhu , A. Lao , R. Zhao , Y. Hu , Y. Zhou , J. Zhou , K. Lin , Y. Xu , Bioact. Mater. 2024, 41, 239. 39149594 10.1016/j.bioactmat.2024.07.016 PMC11324614

[21] Y. Chen , X. Cao , J. Yao , Z. Hu , Y. Luo , G. Li , H. Zhang , K. Wu , Int. J. Biol. Macromol. 2024, 283, 137487. 39579834 10.1016/j.ijbiomac.2024.137487

[22] Y. Zhao , W. Duan , B. Zhu , Y. Chen , Y. Zhu , S. Martin‐Saldaña , Z. Xiao , X. Liu , L. Feng , Y. Ren , Y. Gong , F. Huo , J. Li , Y. Bu , B. Du , L. Zhang , Adv. Funct. Mater. 2024, 35, 2418660.

[23] H. Li , F. Meng , C. Hu , Z. Wu , L. Hao , C. Sun , L. Fang , F. Pan , S. Bian , H. Li , M. Li , B. Liu , X. Zhao , Adv. Sci. 2025, 12, 2500833. 10.1002/advs.202500833 PMC12376595 40317713

[24] L. Quan , Y. Ouyang , W. Liang , Z. Chen , D. Miao , B. Zheng , D. Wu , R. Huang , Adv. Sci. 2025, 12, 04802. 10.1002/advs.202504802 PMC12407365 40576531

[25] J. Kono , M. Ueda , A. Sengiku , S. O. Suadicani , J. T. Woo , T. Kobayashi , O. Ogawa , H. Negoro , Int. J. Mol. Sci. 2022, 23, 5037. 35563427 10.3390/ijms23095037 PMC9102543

[26] C. Brossard , A. C. Lefranc , A. L. Pouliet , J. M. Simon , M. Benderitter , F. Milliat , A. Chapel , Biology 2022, 11, 972. 36101353 10.3390/biology11070972 PMC9311586

[27] I. O. Sherif , J. Cell. Biochem. 2018, 119, 7441. 29775228 10.1002/jcb.27053

[28] X. Chen , Z. Li , X. Ge , X. Qi , Y. Xiang , Y. Shi , Y. Li , Y. Pan , Y. Wang , Y. Ru , K. Huang , J. Shao , J. Shen , H. Li , Adv. Sci. 2024, 11, 2405463. 10.1002/advs.202405463 PMC11615794 39392368

[29] N. Xu , Y. Gao , Z. Li , Y. Chen , M. Liu , J. Jia , R. Zeng , G. Luo , J. Li , Y. Yu , Chem. Eng. J. 2023, 466, 143173.

[30] M. You , Y. Guo , H. Yu , H. Yin , X. Shi , Z. Tang , J. Yang , G. Qin , J. Shen , Q. Chen , Chem. Eng. J. 2024, 500, 157103.

[31] F. Dai , J. Zhang , F. Chen , X. Chen , C. J. Lee , H. Liang , L. Zhao , H. Tan , Adv. Sci. 2024, 11, 2408783. 10.1002/advs.202408783 PMC11633493 39435670

[32] X. Ge , J. Hu , X. Qi , Y. Shi , X. Chen , Y. Xiang , H. Xu , Y. Li , Y. Zhang , J. Shen , H. Deng , Adv. Mater. 2025, 37, 2412240. 10.1002/adma.202412240 39610168

[33] Q. Chen , J. Ma , S. Yu , Y. Su , H. Guo , H. Jiang , H. He , J. Hu , Y. Liu , L. Yao , B. Meng , Z. Yuan , W. Shu , L. Wang , H. Mao , M. Zhang , B. Li , F. Han , Adv. Funct. Mater. 2025, 10.1002/adfm.202507840.

[34] Z. Tian , R. Gu , W. Xie , X. Su , Z. Yuan , Z. Wan , H. Wang , Y. Liu , Y. Feng , X. Liu , J. Huang , Bioact. Mater. 2025, 46, 434. 39850021 10.1016/j.bioactmat.2024.12.014 PMC11755075

[35] T. Zhou , C. Zhu , W. Zhang , Q. Wu , M. Deng , Z. Jiang , L. Peng , H. Geng , Z. Tuo , C. Zou , Front. Immunol. 2025, 16, 1511529. 39917301 10.3389/fimmu.2025.1511529 PMC11799275

[36] J. Zhang , Q. Ge , T. Du , Y. Kuang , Z. Fan , X. Jia , W. Gu , Z. Chen , Z. Wei , B. Shen , Life Sci. Alliance 2025, 8, 202402957. 10.26508/lsa.202402957 PMC11584326 39578076

[37] S. Jiang , T. Jin , T. Ning , Z. Yang , Z. Ma , R. Huo , Y. Cheng , D. Kurdyla , E. Lam , R. Long , A. Moores , J. Li , Nat. Commun. 2025, 16, 6826. 40707461 10.1038/s41467-025-62019-y PMC12290001

[38] J. Yang , S. Wu , M. He , Exploration 2025, 5, 20240349. 40585759 10.1002/EXP.20240349 PMC12199405

[39] L. Zhang , K. Liu , J. Zhou , Y. Zhang , J. Wen , J. He , Y. Zheng , L. Yang , K. Wang , J. Tian , Bioact. Mater. 2025, 52, 687. 40641579 10.1016/j.bioactmat.2025.06.027 PMC12241814

[40] X. Chen , W. Li , Y. Ma , W. Zhang , W. He , F. Ding , S. Guo , D. Geng , G. Pan , Bioact. Mater. 2025, 53, 480. 40755850 10.1016/j.bioactmat.2025.07.038 PMC12314173

