# Supplementary material for: Under‐Urine‐Adhered Supramolecular Hydrogel with Linearly Sustained Quercetin Release Facilitates Hemorrhagic Cystitis Healing via Inflammation Regulation
Source: Adv Sci (Weinh). 2025 Nov 7;13(5):e15003. doi: 10.1002/advs.202515003 (PMC12850167; doi:10.1002/advs.202515003)
Supplement: Supplementary file 1 — Supporting Information [file ADVS-13-e15003-s001.docx]

Supporting Information

Under-urine-adhered Supramolecular Hydrogel with Linearly Sustained Quercetin Release Facilitates Hemorrhagic Cystitis Healing via Inflammation Regulation

Xu Cao^1,2^, Hua Zhang^2^*, Yang Luo^1,2^, Yaoqi Chen^1^, Jie Yao^1^, Renhao Ni^2^, Tong Zhu^2^, Yudong Yao^2^, Jun Chen^3^*, Baolin Guo^4^*, Kerong Wu^1^*

Xu Cao, Yang Luo, Yaoqi Chen, Jie Yao, Kerong Wu

Department of Urology, Translational Research Laboratory for Urology, Ningbo Clinical Research Center for Urological Disease, The First Affiliated Hospital of Ningbo University,

Zhejiang Engineering Research Center of Innovative technologies and diagnostic and therapeutic equipment for urinary system diseases Ningbo, Zhejiang 315010, China.
E-mail: fyywukerong@nbu.edu.cn

Hua Zhang, Renhao Ni, Tong Zhu, Yudong Yao
Research Institute of Smart Medicine and Biological Engineering, Health Science Center, Ningbo University, Ningbo, Zhejiang 315211, China

E-mail: zhanghua@nbu.edu.cn

Jun Chen

Intelligent Polymer Research Institute, Innovation Campus, University of Wollongong, Squires Way, North Wollongong, NSW, 2500, Australia

E-mail: junc@uow.edu.au

Baolin Guo

State Key Laboratory for Mechanical Behavior of Materials, and Frontier Institute of Science and Technology, Xi'an Jiaotong University, Xi'an, Shaanxi 710049, China

E-mail: baoling@mail.xjtu.edu.cn

Experimental Section

***Materials***

HA (Mw 40 kDa–140 kDa), β-cyclodextrin, dimethylformamide triethylamine, acryloyl chloride, quercetin, N-(3-dimethylaminopropyl)-N'-ethylcarbodiimide hydrochloride, N-hydroxysuccinimide, and dopamine hydrochloride were obtained from Aladdin Co., Ltd. (Shanghai, China). Calcein-AM/propidium iodidedouble staining kit, CCK-8, and reactive oxygen species assay kit were supplied by Beyotime Biotech (Shanghai, China) Primer Script™ RT Master Mix kit and TB Green Premix Ex Taq™ kit were provided by Takara (Tokyo, Japan). Artificial urine was provided by Scientific Phygene^®^. Dulbecco's modified Eagle’s medium, PBS, ECM CM-ZY001, Ham’s F12K Medium, fetal bovine serum (FBS), and penicillin/streptomycin (P/S) were purchased from Wuhan Pricella Biotechnology Co., Ltd. The Matrigel was purchased from ABW Company Shanghai, China).

***Synthesis of Ac-β-CD***

Ac-β-CD was synthesized through a nucleophilic substitution reaction between acryloyl chloride and cyclodextrin, following previous studies [1-2]. Initially, 10 g of β-cyclodextrin was dissolved in 150 mL of dimethylformamide. Subsequently, 7 mL of triethylamine was added to the solution. The mixture was placed in an ice bath, maintaining the temperature at approximately 0°C for 10 min. Subsequently, 5 mL of acryloyl chloride was added dropwise to the solution, which was continuously stirred for 12 h at around 0°C. After the reaction, the mixture was filtered to remove any precipitates, resulting in a clear solution. This solution was concentrated to approximately 10% of its original volume using a vacuum rotary evaporator. Ten times the volume of acetone was slowly added to the concentrated solution at room temperature to facilitate the precipitation of Ac-β-CD. The precipitate was washed at least three times with acetone and dialyzed for 24 h using a dialysis membrane with a molecular weight cutoff of 200 Da. Finally, the product was vacuum-dried to obtain a powder. The degree of substitution (DS) of the Ac-β-CD was determined through ^1^H nuclear magnetic resonance analysis using a Bruker Advance 400 MHz spectrometer.

***Synthesis of dopamine modified hyaluronic acid (HADA)***

HADA was synthesized through an amide reaction between HA and dopamine, following a method established in previous studies[3]. HA (1.0 g)was completely dissolved in 100 mL of deionized water under nitrogen atmosphere to prevent dopamine oxidation. To activate the carboxyl groups of HA, 1.48 g of 1-ethyl-3-(3-dimethylaminopropyl) carbodiimide and 0.9 g of N-hydroxysuccinimide were added to the HA solution while stirring vigorously for 20 min. Subsequently, 1.48 g of dopamine was then added to the reaction mixture. The pH of the solution was adjusted to 5.0–5.5 to facilitate the conjugation reaction, which proceeded for 8 h. The resulting solution was transferred into dialysis bags with a molecular weight cutoff range of 8000–14000 Da, and dialysis was performed in deionized water for 48 h to remove any unreacted reagents and salts. The dialyzed solution was then freeze-dried to obtain HADA. The chemical structure of HADA was verified using FTIR spectroscopy (Thermo Fisher Nicolet IS5).

***^1^H nuclear magnetic resonance (^1^H-NMR) analysis of HADA***

The quantification of dopamine groups grafted onto HA was determined through ^1^H-NMR analysis using a Bruker Advance 400 MHz spectrometer. For the analysis, approximately 50 mg of freeze-dried HADA conjugate was dissolved in 600 μL of deuterium oxide (D₂O), and the spectrum was acquired after complete dissolution. The degree of substitution (*DS*) was calculated according to the following equation:

$$DS\left( \% \right)=\frac{A_{H}(6.6-7.2)/3}{A_{H}(3.0-4.0)/10}\times100$$

where *A_H_*(6.6-7.2 ppm) is the signal integral area for the three aromatic protons of the catechol moiety, and *A_H_*(3.0-4.0) is the integral for the ten backbone protons of HA.

***Preparation of HGCQ hydrogels***

The HGCQ hydrogels were prepared by uniformly mixing gelatin, Ac-β-CD, HADA, and quercetin through a one-pot method, followed by photoinitiated Ac-β-CD polymerization. Initially, 4 g of gelatin, 5 g of Ac-β-CD, and 0.1 g of photo-initiator lithium phenyl-2,4, 6-trimethylbenzoylphosphonate were added directly to 50 mL of PBS. The mixture was heated at 40 °C and stirred using a magnetic stirrer until the gelatin/Ac-β-CD was completely dissolved, resulting in a homogeneous solution. Subsequently, the desired concentrations of HADA (0.5%, 1.0%, or 1.5% (w/v)) and 1.0 mg/mL of quercetin were incorporated into the solution, and mixing was continued until the components were fully integrated. The precursor solution was centrifuged for 5 min to remove the air bubbles. The resulting mixtures were subsequently poured into transparent PVC molds and exposed to 405 nm ultraviolet light (10 mW/cm ^2^) for 60 s to form hydrogels. To optimize the concentrations of quercetin within the hydrogels, additional formulations were prepared with quercetin concentrations of 0.5 mg/mL, 1.0 mg/mL, and 1.5 mg/mL, combined with 8% gelatin, 10% Ac-β-CD, and 1.5% HADA. Additionally, as controls, gelatin/Ac-β-CD hydrogels and gelatin/Ac-β-CD hydrogels loaded with 1.0 mg/mL of quercetin were prepared under the same conditions.

***Isothermal titration calorimetry (ITC) test***

The interactions between the guest polymer and the main-chain polymer were characterized by ITC using a TA Instruments Nano ITC calorimeter at 25.00 ± 0.01°C in single-injection titration mode[4]. To avoid gelation or significant viscosity increase at high polymer concentrations, the experiments were conducted using 0.3 mM gelatin and a mixture of 0.3 mM gelatin with 0.2 mM HADA in PBS buffer (pH 7.4). The host molecule Ac-β-CD was maintained at 5.0 mM to ensure cavity excess. Single-point titration was performed with a stirring speed of 200 rpm, an injection volume of 2 μL, and an initial delay time of 300 s

***Injection force testing***

The injection forces required for the hydrogel precursors were measured using a standard mechanical testing machine equipped with a 200 N sensor (Sasck, CMT1104). Hydrogel precursors 0.5 mL were loaded into 1-mL syringes fitted with 26-G needles, which were then positioned within a sleeve for stability during testing. The average injection force and the corresponding displacement-force curve were recorded at a loading speed of 40 mm/min.

***CCK8 assay***

The cytotoxicity of the HGCQ hydrogel loaded with quercetin was evaluated using a Cell Counting Kit-8 (CCK-8) assay in a Transwell co-culture system. Briefly, SV-HUC-1 cells were seeded in the lower chamber of a 24-well plate at a density of 8×10⁴ cells per well. Simultaneously, 500 mg of hydrogels containing quercetin at concentrations of 0.5, 1.0, and 1.5 mg/mL were placed in the upper chamber. The co-culture was maintained for 72 hours. At 24, 48, and 72-hour time points, cell proliferation and viability were assessed. Following the manufacturer's protocol, the CCK-8 reagent was added to the cells, and after incubation, the absorbance was measured at 450 nm using a multifunctional microplate reader (Infinite M200 Pro, TECAN).

To quantify the absolute number of viable cells, a standard curve was generated. Specifically, 500 to 8000 cells per well were seeded in a 96-well plate (n=3), allowed to adhere for 6 hours, and then subjected to the CCK-8 assay. The absorbance data were linearly fitted using GraphPad Prism 9 software. The resulting standard curve equation was then used to convert the absorbance readings from the experimental groups into the precise number of viable cells at each time point. The cell viability under different treatment conditions was normalized to that of the control group.

***Swelling tests***

Hydrogel samples with a diameter of 8 mm and a height of 4 mm were prepared for swelling measurements. The initial weight (W_0_) of each fresh hydrogel sample was recorded before immersion in an artificial urine solution held at 37°C. Following immersion, the swollen weight (W_t_) of the hydrogels was measured at 6, 12, 24, 48, and 72 h. All experiments were conducted in triplicate. The swelling ratios of the hydrogels were calculated using the following formula:

$$Swelling ratio \left( \% \right)=\frac{W_{t}-W_{0}}{W_{0}}\times100$$

***In-vitro degradation of hydrogels***

For the in vitro degradation measurements, cylindrical hydrogel samples with a height of 4 mm and a diameter of 8 mm were fabricated. The freshly prepared samples were lyophilized to determine their initial dry weights (W_0_). The hydrogels were then immersed in artificial urine to simulate the fluid environment of the bladder, with the artificial urine replaced every 6 h to mimic the bladder voiding and refilling cycle. At designated time intervals, the samples were removed, lyophilized, and weighed to assess the remaining hydrogel mass (W_t_). For comparison, parallel hydrogel samples were immersed in PBS as control. All experiments were conducted in triplicates. The remaining hydrogel mass was calculated using the following formula:

$$Degradation ratio \left( \% \right)=\frac{W_{t}}{W_{0}}\times100$$

***Morphological characterization by scanning electron microscopy (SEM)***

The morphological evolution of the HGCQ hydrogel during swelling and degradation in artificial urine was characterized using SEM. Hydrogel samples were prepared at various time points (0, 6, 12, 24, 48, 72, 96, and 120 hours) during the incubation process. At each predetermined time point, the samples were carefully retrieved and immediately frozen in liquid nitrogen for 10 minutes to preserve their instantaneous swollen and degraded structure. The frozen samples were then transferred to a freeze-dryer (Scientz-30N-pro) and lyophilized for 48 hours to completely remove the water content. The lyophilized hydrogels were subsequently fractured to expose their internal cross-sectional morphology. The fragments were mounted on aluminum stubs using conductive carbon tape and sputter-coated with a thin layer of gold to enhance surface conductivity. The microstructures of the samples were then observed under a SEM (Pharos G2, Phenom) at an accelerating voltage of 5 kV. Representative images were captured to analyze the changes in pore size and overall structural integrity

***Under-urine adhesion measurement***

The adhesion stability of the HGCQ hydrogel to wet bladder tissue in urine was evaluated using a high mechanical shear method. After the HGCQ hydrogel was adhered to the tissue, a biological adhesive was used to securely attach the porcine bladder to a stirring paddle, which was then placed in artificial urine and subjected to a shear stability test at 300 rpm for 60 min.

***Burst pressure test***

Burst pressure was tested following a previously established protocol [5-6]. A 3 cm diameter section of the porcine bladder was excised and secured onto a measurement apparatus connected to a syringe pump. A 5 mm diameter circular hole was created in the bladder membrane, which was maintained under moist conditions throughout the experiment. Subsequently, 500 μL of the HGCQ hydrogel precursor solution was injected into the defect and allowed to crosslink in situ, forming a hydrogel patch with a final thickness of approximately 4 mm covering the puncture site. To simulate a dynamic urinary environment, the entire assembly was immersed in a 3 L beaker containing artificial urine maintained at 37 °C under continuous stirring at 700 rpm. The artificial urine was replaced every 5 h to maintain consistent physicochemical conditions. Pressure was gradually applied via the syringe pump until hydrogel failure occurred. The burst pressure, defined as the maximum pressure recorded immediately before a sudden pressure drop, was used to quantify the adhesive strength of the hydrogel under flow conditions. All experiments were performed in triplicate to ensure statistical reliability.

***Adhesive mechanical testing of hydrogels***

The adhesive mechanical performance of the hydrogels on porcine bladder tissue was assessed using 90-degree peel testing, lap-shear testing, and tensile testing (200 N load-cell, CMT1104, Sasck). All experiments used porcine bladder tissue and were conducted at a constant speed of 3 mm/min. In the 90-degree peel test, a hydrogel square measuring 1.5 cm × 1.5 cm was photocured in situ between a glass slide and the bladder tissue (2 cm × 8 cm). The interfacial toughness was calculated by dividing the plateau force by the sample width. In addition, the shear strength of the hydrogels was evaluated using a lap-shear test. Porcine bladder tissue was sectioned into squares measuring 1.5 cm × 1.5 cm and secured to the end of a glass slide with a bioadhesive. A volume of 200 μL of hydrogel precursor solution was applied to the tissue and subsequently covered with another glass slide. The hydrogels were then photocured for 60 s. The shear strength was calculated by dividing the maximum force obtained from the lap-shear test by the adhesive area of the sample. Furthermore, the tensile strength of the hydrogel adhered to the porcine bladder tissue was determined by tensile testing and calculated by dividing the maximum force from the tensile test by the adhesive area of the samples.

***Rheological characterization of hydrogels***

The rheological properties of GC, GCQ, and HGCQ hydrogels were evaluated using a dynamic rheometer (Discovery HR20, TA Instrument) equipped with a 20-mm diameter plate and a 1000-μm gap. To investigate the photo-crosslinking process, the hydrogel solutions were exposed to UV light for 6 min during oscillatory time sweeps at a frequency of 10 rad/s and a strain of 1% Storage and loss moduli were continuously recorded to monitor the kinetics of the crosslinking process. Subsequently, the storage and loss moduli were assessed over a frequency range of

10^-1^ to 10^2^ rad/s while maintaining a constant strain of 1%.

***Hemorrhage Model***

Three hemorrhage models were employed to evaluate the hemostatic efficacy of HGCQ hydrogel: the liver hemorrhage model, the femoral vein hemorrhage model, and the tail truncation hemorrhage model. In the liver hemorrhage model, rats were anesthetized, and a longitudinal abdominal incision was made to expose the liver. A 10 mm horizontal and vertical incision was created in the middle of the left hepatic lobe to induce hemorrhage. Hemostasis was achieved using gelatin hemostatic sponges and HGCQ hydrogel, with hemostasis time and blood loss recorded. Blood loss (ΔM) was determined by weighing pre-weighed filter paper (M0) placed under the liver and comparing it to the weight of the filter paper post-hemostasis (Mt). In the femoral vein hemorrhage model, the right hind limb of anesthetized rats was incised to expose the femoral vein. Hemostatic forceps were applied to clamp the vein proximally at the abdominal end and the toe end. A 5 mm incision was made in the mid-portion of the femoral vein to induce hemorrhage. Hemostasis was conducted using gelatin hemostatic sponges and HGCQ hydrogel, with the forceps released simultaneously. The hemostasis time was recorded, and blood loss was calculated by weighing pre-weighed filter paper (M0) placed under the incision and comparing it to the weight post-hemostasis (Mt). In the tail truncation hemorrhage model, the tails of anesthetized rats were transected 5 cm from the tip, allowing free bleeding for approximately 5 seconds. Hemostasis was then performed using gelatin sponges and HGCQ hydrogel, and the hemostasis time was recorded. Blood loss was quantified by weighing pre-weighed filter paper (M0) placed at the wound site and comparing it to the weight after hemostasis (Mt).The blood loss in each model was calculated using the formula:

$$\Delta M=M_{t}-M_{0}$$

This systematic approach allowed for a comprehensive evaluation of the hemostatic performance of HGCQ hydrogel across different types of hemorrhage scenarios.

***Evaluation of hemocompatibility***

Hemolysis tests were performed in accordance with International standard ISO 10993-5 [7]. Cylindrical hydrogel samples, each with a height of 3 mm and a diameter of 9 mm, were incubated in diluted blood from New Zealand rabbit blood (diluted ten-fold with saline) at 37°C for 60 min. Deionized water-diluted and saline-diluted blood served as positive and negative controls, respectively. The diluted blood was centrifuged at 750 *g* for 5 min, and the supernatant was collected. Absorbance of the supernatant was measured at 545 nm. The hemolysis ratio of the hydrogels was calculated using the following formula:

$$Hemolysis ratio (\%)=\frac{A_{h}-A_{NC}}{A_{PC}-A_{NC}}\times100$$

where A_h_, A_PC_, and A_NC_ represent the absorbance values of the hydrogel, positive control, and negative control groups, respectively.

***In-vitro cytocompatibility***

Rat bladder smooth muscle cells were sourced from the Health Science Center of Ningbo University and seeded onto the surface of the HGCQ hydrogels at a density of 5×10^4^ cells/mL in 24-well plates. Cells were cultured in DMEM/F12 medium supplemented with 10% FBS and 1% penicillin/streptomycin. After 24 h of incubation, the cells were fixed and stained with phalloidin for F-actin visualization and DAPI for nuclear staining, enabling the observation of cellular morphology. Confocal laser scanning microscopy (CLSM, STELLARIS 5, Leica) was employed to capture the detailed cellular structures.

***Quercetin release assessment***

The characteristic peak of quercetin was identified at 385 nm by using a UV spectrophotometer (LAMBDA 850+). Standard solutions with concentrations of 7.5, 15, 30, 60, 90, and 180 mg/L were prepared and used to construct standard curves. Dialysis was employed to evaluate quercetin release from the hydrogels using samples collected at predetermined time points. The amount of released quercetin was calculated using a standard curve. To ensure accuracy and reliability, each experimental group was tested in triplicates. This systematic approach allowed for a comprehensive assessment of the quercetin release profile.

***HADA release assessment***

The characteristic absorption peak of HADA was identified at 280 nm using UV spectrophotometry (LAMBDA 850+), attributed to the specific absorption of the catechol functional group. A standard curve was constructed using standard solutions with concentrations of 15.625, 31.25, 62.5, 125 and 250 mg/L. The release of HADA from the hydrogels was evaluated through dialysis methodology, with samples collected at predetermined time intervals. The concentration of released HADA was quantified using established standard curves. All measurements were performed in triplicate to ensure statistical reliability and reproducibility.

***DPPH scavenging ability assessment***

The DPPH scavenging abilities of GC, GCQ, and HGCQ gels were evaluated by co-incubating them with 1 mM DPPH in a light-protected environment for 72 h. At specified time intervals, the supernatant was collected and analyzed at 517 nm using a multifunctional microplate reader (Infinite M200 Pro, TECAN). DPPH scavenging ability was calculated using the following equation:

$$DPPH scavenging rate (\%)=\frac{A_{0}-A_{x}}{A_{0}}\times100$$

where *A*_0_ represents the absorbance of the control group and *A_x_* is the absorbance of the experimental group.

***Evaluation of ROS scavenging performance in vitro***

The in vitro ROS scavenging performance was assessed by live/dead cell staining. SV-HUC-1 cells were plated in 24-well culture plates at a density of 5.0 × 10^4^ cells per well and incubated for 24 h in complete culture medium, prepared with Ham’s F12K medium and supplemented with 600 µM H_2_O_2_ as the control group. The experimental groups included the same concentration of hydrogen peroxide combined with 48-hour extracts of GC, GCQ, and HGCQ. The SV-HUC-1 cells were stained with a Calcein-AM/PI staining kit for 30 min in the dark at 37 °C. Images were captured using CLSM to evaluate the cell viability and ROS-scavenging effectiveness.

***Antibacterial activity of the hydrogels***

The antibacterial efficacy of the hydrogels was quantitatively evaluated using a plate spreading method [8]. *E. coli* and *S. aureus* were selected as the model bacteria. The bacteria solution (10^6^ CFU·mL^−1^) was treated with GC, GCQ, and HGCQ hydrogel, respectively. After incubation at 37°C for 12 h, 100 µL of the diluted bacteria solutions were spread onto solid LuriaBertani plates. The plates were then incubated at 37°C for an additional 12 h. CFUs on each plate were photographed using a digital camera, and the colonies were counted to assess bacterial viability.

***Tube formation assay***

The effect of the HGCQ hydrogel on angiogenesis was assessed using a tube formation assay using a Transwell system. Matrigel was added to the lower chamber of the Transwell (200 μL per well) and incubated at 37 °C for 30 min to allow for hydrogel formation. Green fluorescent protein-labeled HUVECs, provided by the Tissue Engineering Laboratory at Ningbo University, were seeded onto the Matrigel surface at a density of 5 × 10^5^ cells per well and maintained in complete ECM culture medium. After allowing the cells to adhere and spread on the Matrigel for 4 h, the culture medium was promptly replaced with hydrogel leachate prepared in a serum-free medium. After 24 h of coculture, the cells were observed under CLSM, and tube formation was analyzed using the angiogenesis analysis plugin of the ImageJ software.

***Scavenging LPS-induced ROS in raw 264.7 cells***

The intracellular ROS-scavenging ability of the HGCQ hydrogels was measured using an ROS assay kit (Beyotime, S0033). Raw 264.7 cells (5×10^4^ cells/well) were seeded in confocal microscopy dishes and cultured overnight at 37 °C in a 5% CO_2_ atmosphere. Cells were treated with DMEM supplemented with 10% FBS and 1% penicillin/streptomycin containing 500 ng/mL LPS for 12 h. Afterward, the media were gently removed, and the cells were exposed to the leachate obtained from hydrogels extracted in DMEM medium for 24 h. Subsequently, the cells were treated with DCFH-DA (500 µL, 1 µL/mL) in serum-free medium and incubated for 30 min. Hoechst 33342 (1:100) was added, and the cells were incubated for an additional 30 min. After multiple washes with PBS, fluorescence images were captured using CLSM, and the fluorescence intensity was quantified using the ImageJ software.

***Phenotype assessment of macrophage polarization***

Macrophage RAW264.7 cells were seeded in confocal microscopy dishes and cultured in DMEM supplemented with 10% FBS and 1% penicillin/streptomycin for 12 h, followed by activation with LPS (500 ng/mL) for an additional 12 h. After LPS activation, macrophages were incubated with different hydrogels for 24 h and subsequently fixed with 4% paraformaldehyde. The fixed cells were then incubated with primary antibodies against iNOS and CD206 at 4 °C for 12 h. Afterward, the cells were incubated with the corresponding fluorescent-labeled secondary antibodies for 30 min. Cell nuclei were stained with DAPI. Cell morphology was assessed by sequential staining with TRITC-phalloidin for 45 min, followed by DAPI for 15 min, in accordance with the manufacturer’s protocols. These cells were observed using CLSM, and the fluorescence intensity was quantified using the ImageJ software. Cytokine secretion was examined by using Mouse TNF-α (KE10002, Proteintech) following the manufacturer’s protocols.

***RNA extraction and quantitative PCR (qPCR) analysis***

The expression levels of the inflammatory genes, including IL-1β, TNF-α, Arg-1, and IL-4, were quantified using a qPCR assay. Primer sequences used for this analysis are listed in **Table S1**. RAW 264.7 cells treated with LPS, LPS+GC, LPS+GCQ, or LPS+HGCQ were processed for RNA extraction. Total RNA was extracted using RNAiso Plus reagent and reverse transcribed into cDNA using PrimeScript RT Master Mix, according to the manufacturer’s instructions (TaKaRa, Japan). qPCR was performed using Power SYBR Green PCR Master Mix (Applied Biosystems, USA) on a LightCycler 4800II (Roche, Switzerland). The cycling conditions included an initial denaturation at 95°C for 10 min, followed by 40 cycles of 95°C for 20 s, annealing at an optimal temperature for 30 s, and extension at 72°C for 30 s. A final melting curve was generated from 60°C to 95°C with a heating rate of 0.1 °C/s. Relative fold changes of the target genes were calculated using the 2^–ΔΔCT^ method, with glyceraldehyde-3-phosphate dehydrogenase expression as the reference.

***RNA sequencing analysis***

RAW 264.7 cells treated with LPS and LPS+HGCQ were collected (n = 3) and disrupted using TRIzol reagent for mRNA extraction. High-quality RNA was used to construct a sequencing library. Transcriptome sequencing was performed by OE Biotech Co. Ltd. (Shanghai, China). Sequencing results were analyzed and visualized using the CNSknowall platform (https://cnsknowall.com), a comprehensive web service for data analysis.

***Evaluation of HGCQ hydrogel for treating hemorrhagic cystitis in Sprague–Dawley rat model***

All animal procedures were conducted in strict accordance with the national and international guidelines for animal experimentation, with ethical approval obtained from the Animal Ethics and Welfare Committee of Ningbo University (AEWC-NBU20250343). A model of hemorrhagic cystitis in SD rats was established by intraperitoneal injection of 150 mg/kg cyclophosphamide and maintained for 24 h, following protocols from our previous studies. After 24 h, the rats were anesthetized with isoflurane and stabilized. An F3-sized urinary catheter coated with liquid paraffin as a lubricant was inserted through the urethra into the bladder. Proper catheter placement was confirmed based on the appearance of urine flow. Gentle abdominal pressure was applied to empower the bladder. Subsequently, 0.25 mL of the precursor solution was injected into the bladder using a syringe. After a 5-minute maintenance period, the excess hydrogel solution (approximately 0.15 mL) was expelled by applying pressure to the abdomen. Following the removal of the syringe, a fiber optic cable connected to a 405-nm UV light source was inserted through the catheter into the bladder, and the light source was activated for 60 s to promote the formation of a hydrogel adhesive layer on the bladder wall. Finally, the fiber optics and catheter were carefully removed. All steps were performed gently to minimize potential trauma to the urinary tract. The operational processes were monitored in situ using an Esaote MyLab ultrasound machine.

***SEM observation***

The interfacial integration of the hydrogel adhesive on the bladder surface was observed using scanning electron microscopy (SEM, Phenom Pharos G2). Fresh samples were fixed in 2.5% glutaraldehyde for 24 h and then rinsed thrice in pure water, with each wash lasting 20 min. The samples were subsequently dehydrated using a graded ethanol series (30%, 50%, 70%, 80%, 85%, 90%, 95%, and 100%), and each concentration was maintained for 30 min. To facilitate the rapid evaporation of alcohol, the samples were then subjected to heating in an oven at 40°C. Prior to SEM examination, the samples were coated with gold-palladium for 3 min.

***Hematuria observation***

Rat urine was collected to evaluate the therapeutic effects of the HGCQ hydrogel adhesive on hemorrhagic cystitis. Urine samples were obtained via catheterization at 6, 12, 24, 48, 72, and 96 h post-treatment. The collected urine was stored in sterile EP tubes and immediately photographed for documentation. A hemocytometer was used to count the blood cells in the urine to assess the degree of hematuria and accurately determine the number of red blood cells.

***HE and immunofluorescence staining***

Bladder specimens were collected at predetermined time points and euthanized. Samples were fixed in 4% paraformaldehyde for 24 h and subsequently dehydrated in a 30% sucrose solution. Dehydration was considered complete when the sample sank to the bottom. The samples were then embedded in OCT compound. Cryosections were prepared at a thickness of 8 μm using a Leica CM1950 cryostat. Prior to H&E staining, the sections were rinsed with distilled water to remove the OCT compounds. The sections were then stained in a hematoxylin solution for approximately 10 min, followed by rinsing with running water to remove excess dye. Next, the sections were immersed in an eosin solution for approximately 2 mins, followed by rinsing under running water. Finally, the sections were dehydrated, cleared, and mounted for observation and photography to assess the histological changes.

Immunofluorescence staining was performed on selected sections to evaluate the degree of inflammation in the bladder tissue. The sections were washed with PBS to remove residual fixatives. To minimize non-specific binding, the sections were blocked with a PBS solution containing 5% normal goat serum at room temperature for 1 h. Next, the diluted primary antibody (**Table S2**) was applied to the sections, ensuring they were fully covered, and incubated overnight at 4°C. Following incubation, the sections were washed thrice with PBS for 5 min each to remove any unbound primary antibodies. Subsequently, a fluorescence-conjugated secondary antibody (**Table S2**) was added and incubated at room temperature for 1 h in the dark. After incubation, the sections were washed thrice with PBS for 5 min each. Finally, the sections were mounted with fluorescent mounting medium containing DAPI to label the cell nuclei. The sections were observed and imaged using the CLSM (Leica STELLARIS 5) to assess the expression and localization of specific proteins.

***Statistical analysis***

All statistical analyses were conducted using GraphPad Prism 9 software, with results presented as mean ± standard deviation (SD). Each experiment was repeated at least three times using biologically independent samples. Unpaired t-tests were employed to compare two groups, while one-way ANOVA followed by Tukey’s post-hoc test was utilized, followed for comparisons among multiple groups. Statistical significance was defined as *p < 0.05, **p < 0.01, and ***p < 0.001.


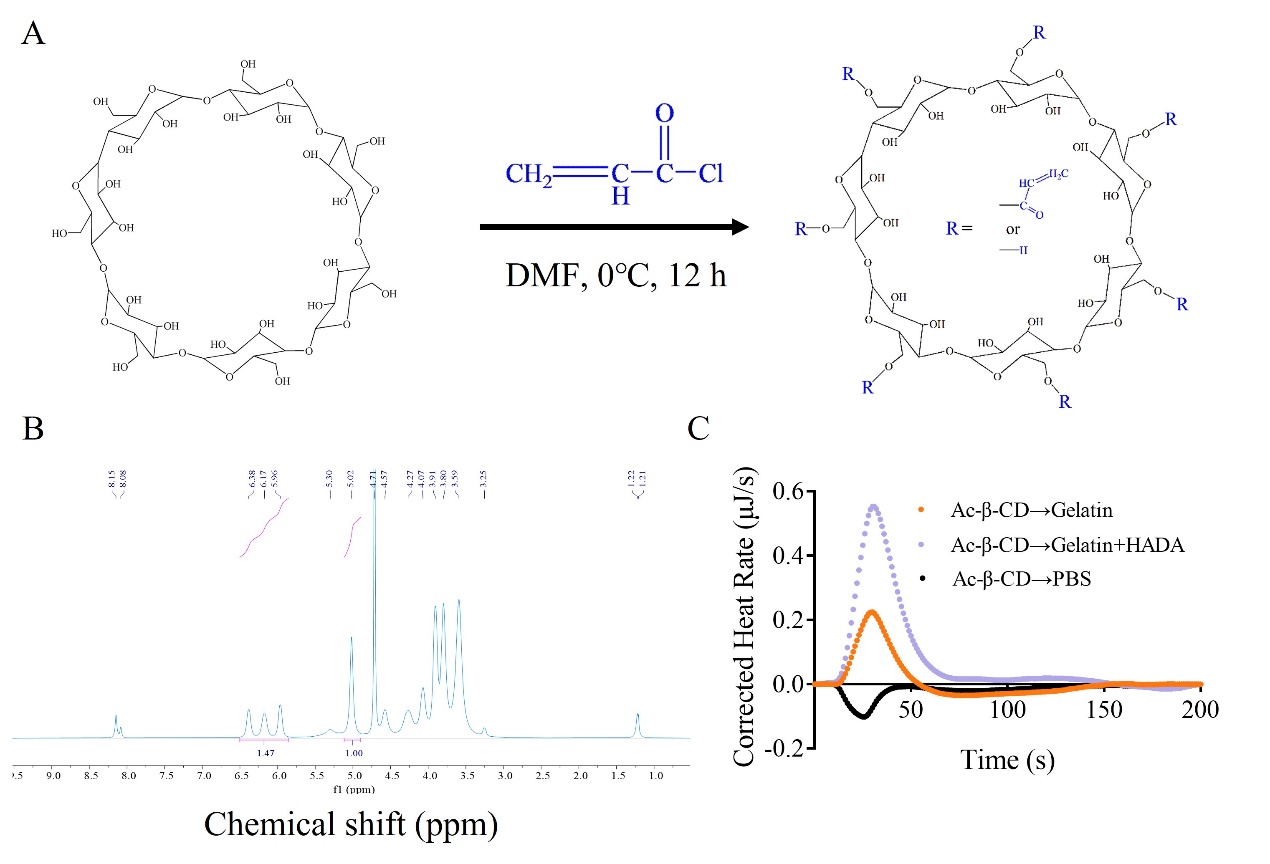


**Figure S1. (**A)Synthetic route of ac-β-CD and (B)^1^H-NMR spectrum of ac-β-CD**.** The degree of substitution of β-CD was nearly 3.0. (C) Comparison of the corrected heat rates for Ac-β-CD interactions with different solutions: Gelatin, Gelatin+HADA, and PBS.


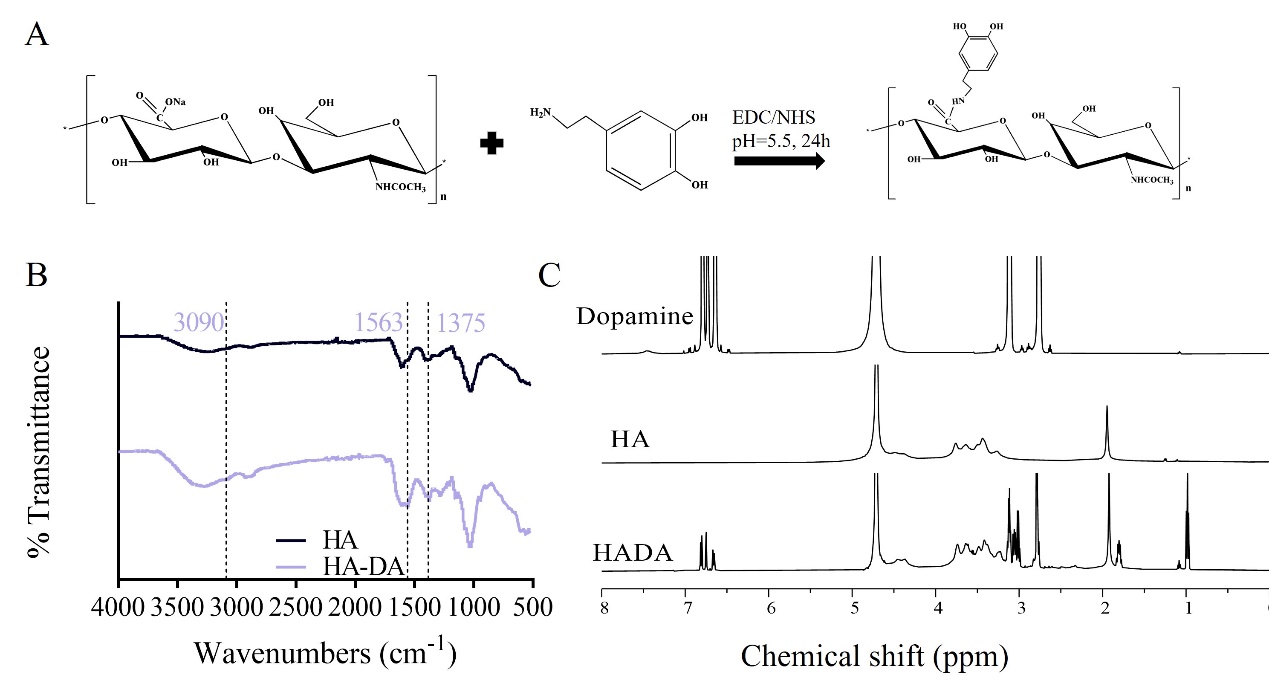


**Figure S2.** (A)Synthetic route of HADA by grafting DA onto HA using EDC/NHS as

activators. (B)FITR spectra and (C)1H NMR confirmed the successful synthesis of HADA.


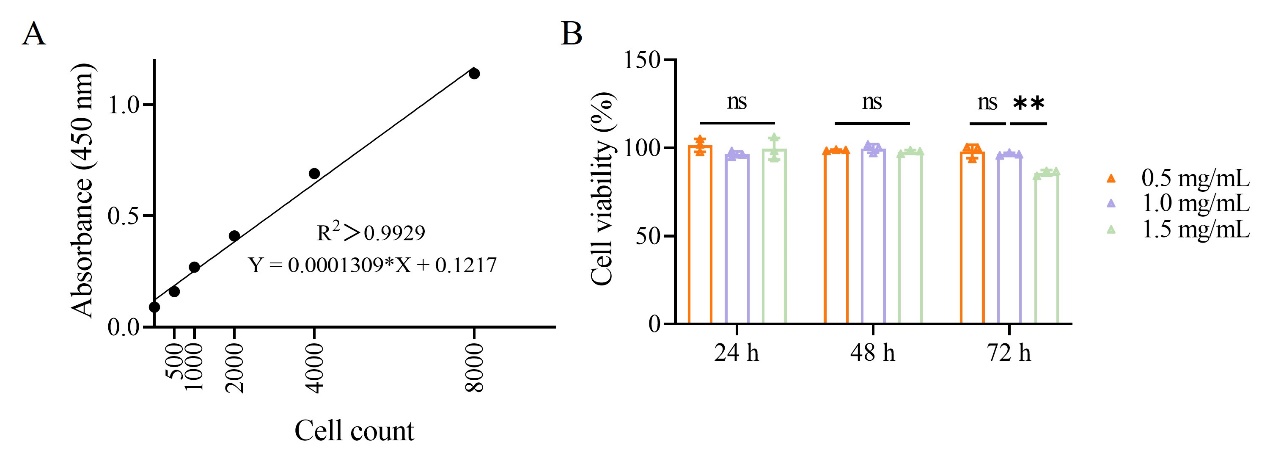


**Figure S3.** Quantitative analysis of SV-HUC-1 cell viability. (**A**) Standard curve generated from known cell densities using the CCK-8 assay. (**B**) Cell viability quercetin-loaded hydrogels under different treatment conditions, normalized to the control group.


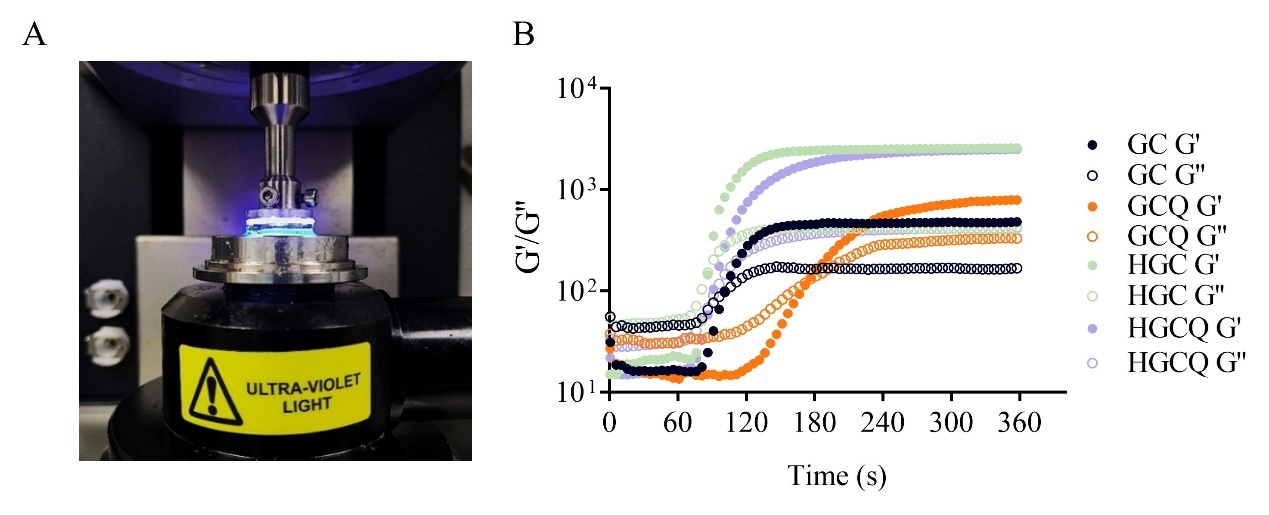


**Figure S4.** Gelation characterization of the hydrogel. (**A**) Image of the rheological measurement under UV irradiation. (**B**) Rheological time-sweep profiles (storage modulus G’ and loss modulus G’’) recorded during photopolymerization, demonstrating the sol-gel transition.


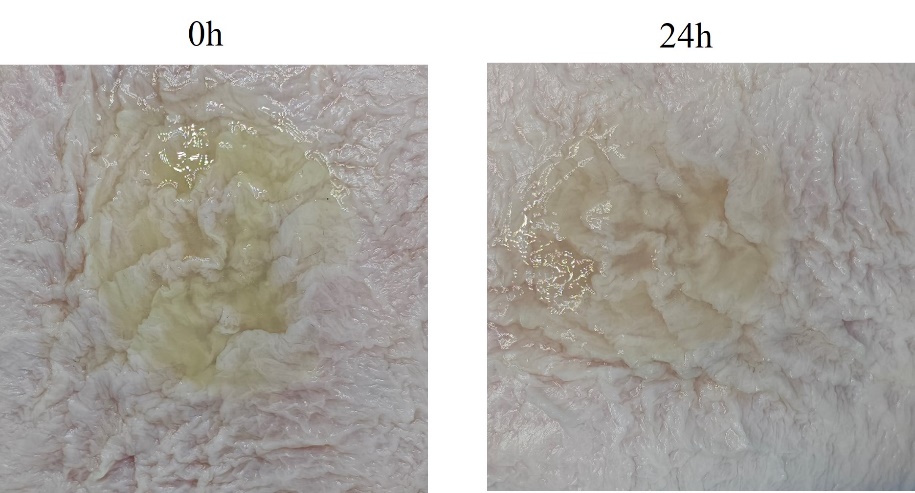


**Figure S5.** Photographs of the hydrogel adhering to the pig bladder.


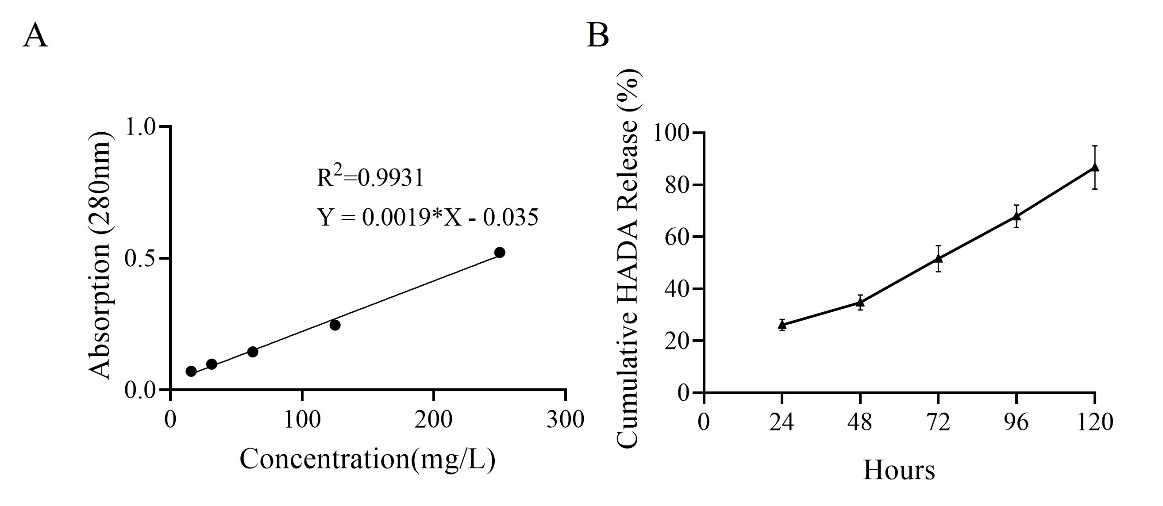


**Figure S6.** (A)Standard curve of HADA and (B)the release curve in artificial urine.


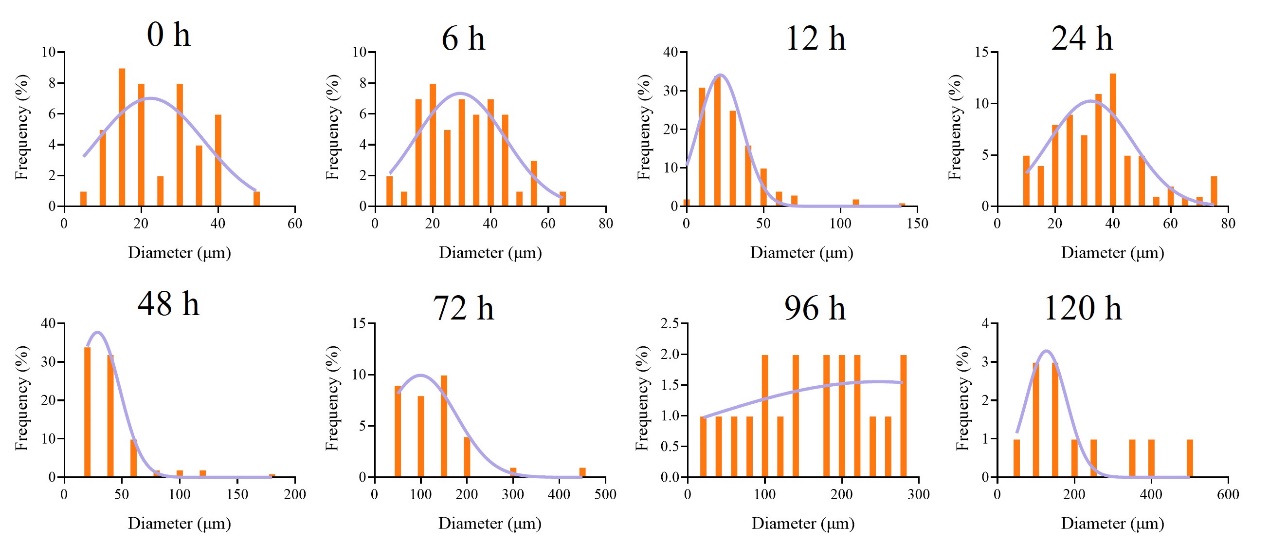


**Figure S7.** The pore diameter distribution of HGCQ hydrogel in artificial urine at given time point.


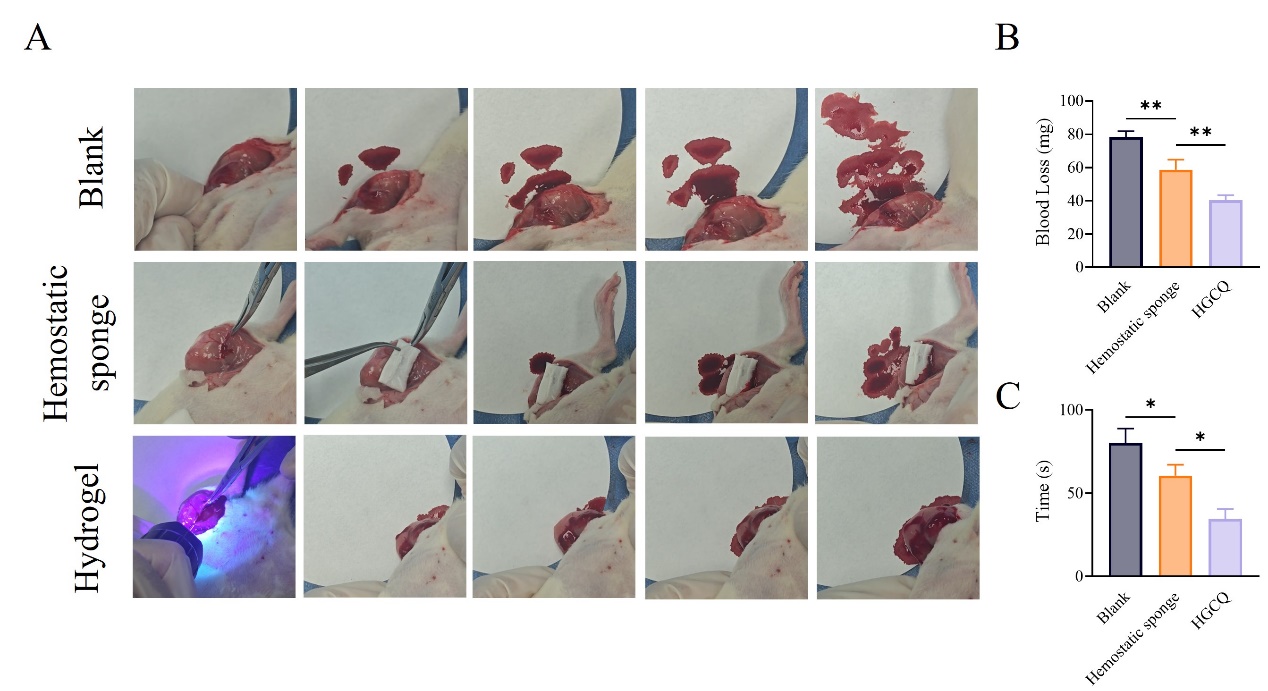


**Figure S8.** In vivo hemostatic efficacy of HGCQ in rat models. (A) Representative images of the tail bleeding model treated with HGCQ, with Blank and Hemostatic sponge as controls. (B) Blood loss following and (C) hemostatic time following treatment with HGCQ hydrogel.


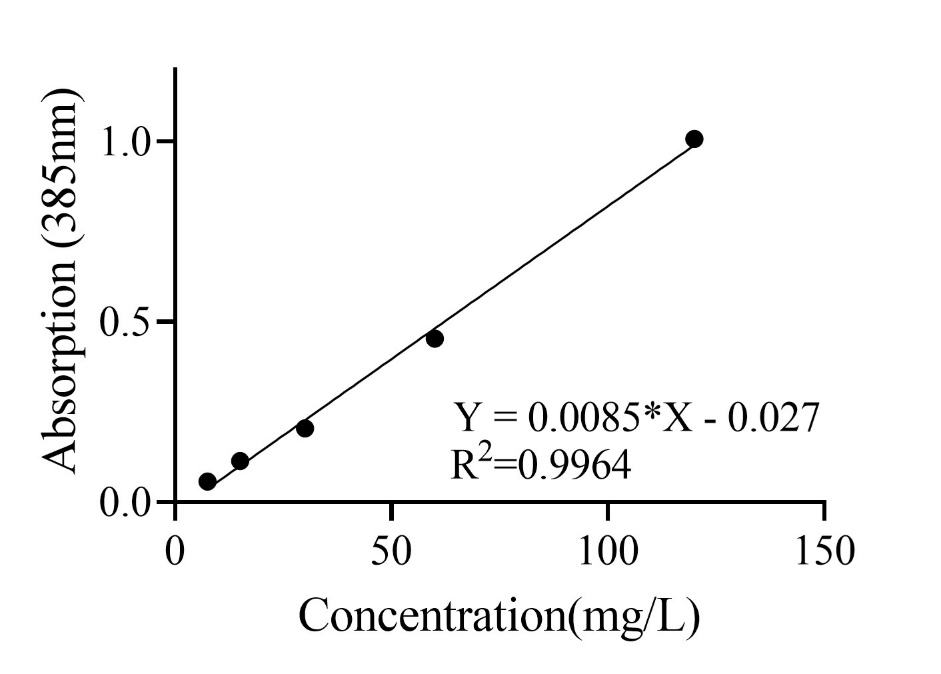


**Figure S9.** Standard curve of quercetin.


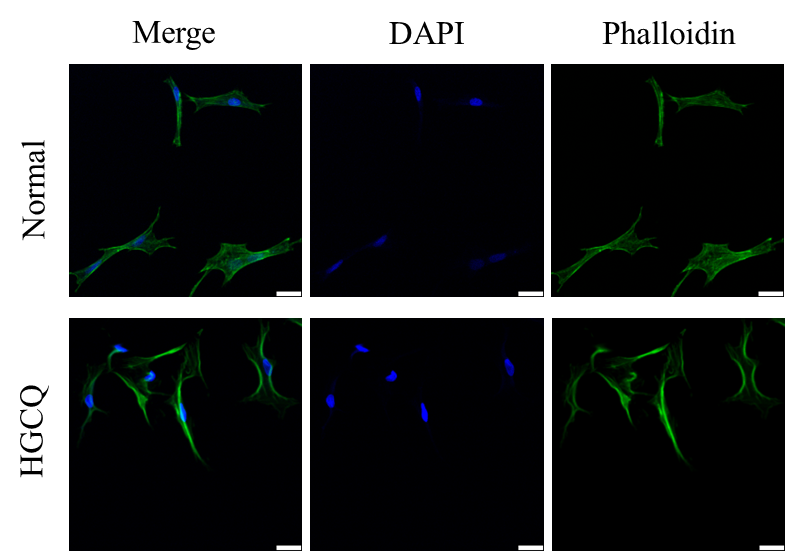


**Figure S10.** Microscopic images of cytoskeleton staining (Scale bar= 25μm).


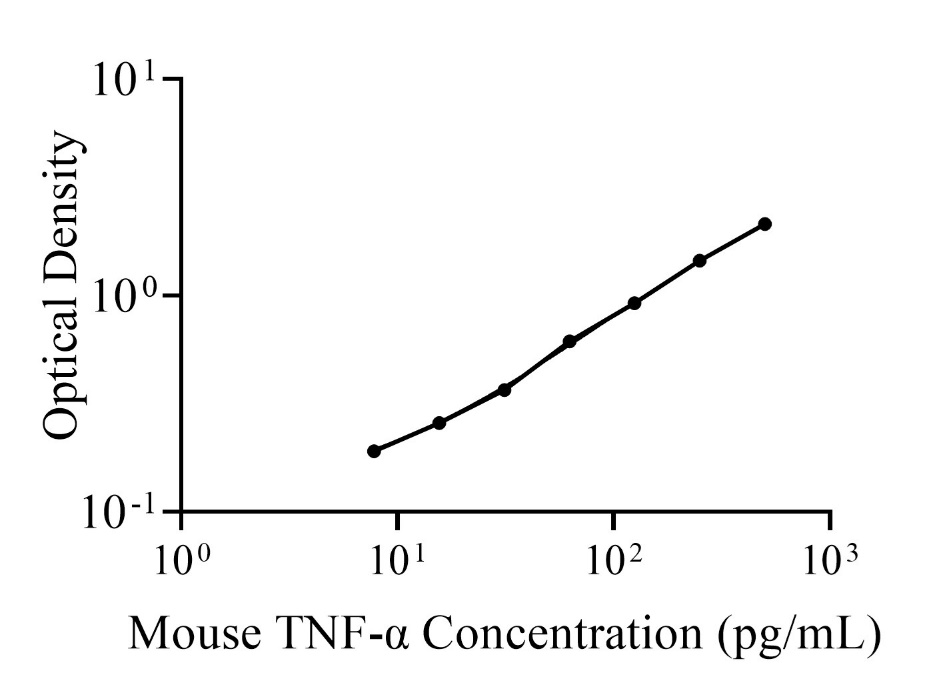


**Figure S11.** Standard curve of mouse TNF-α concentration.


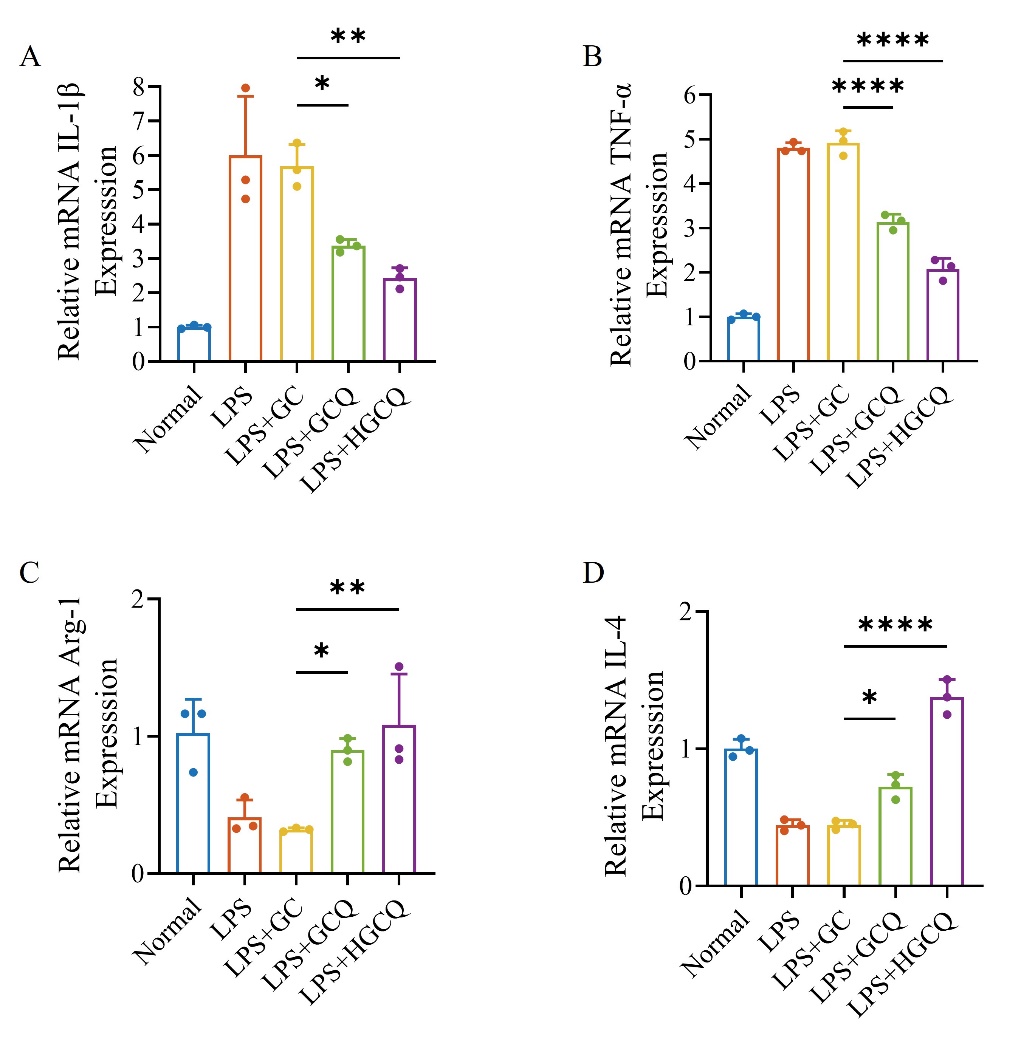


**Figure S12.** Relative mRNA expression of M1-related IL-1β (A) and TNF-α (B) genes, and M2-related Arg-1 (C) and IL-4 (D) genes of macrophages on day 1 measured by RT-qPCR.


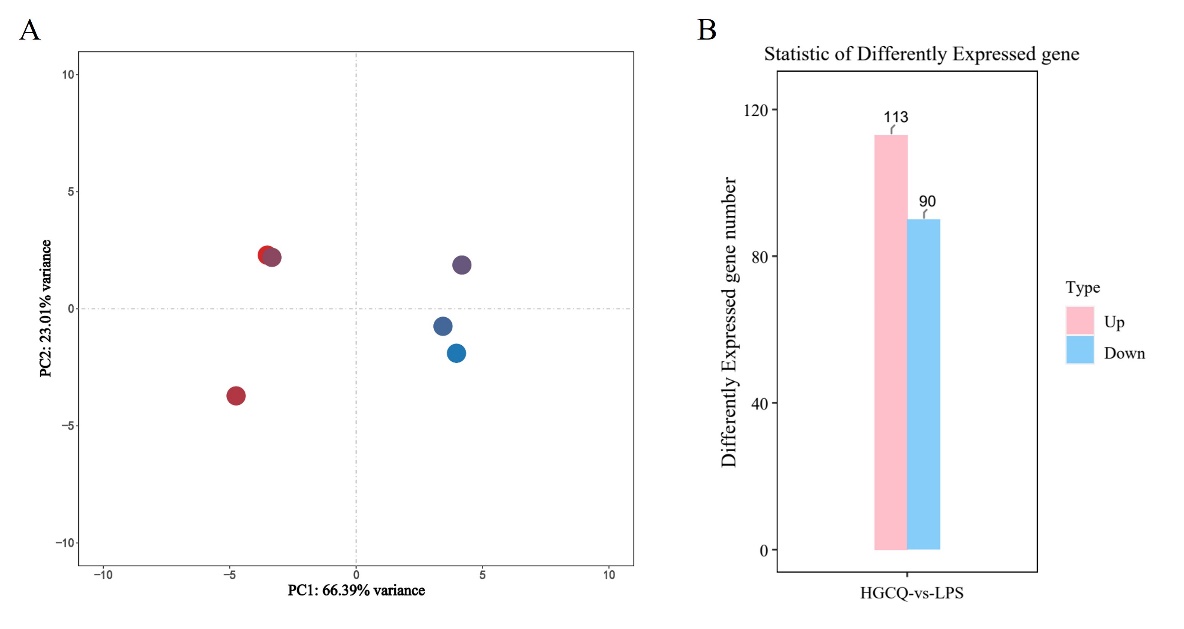


**Figure S13.** (A) Principal component analysis conducted on three groups, with each symbol denoting a single sample and (B) differentially expressed genes.


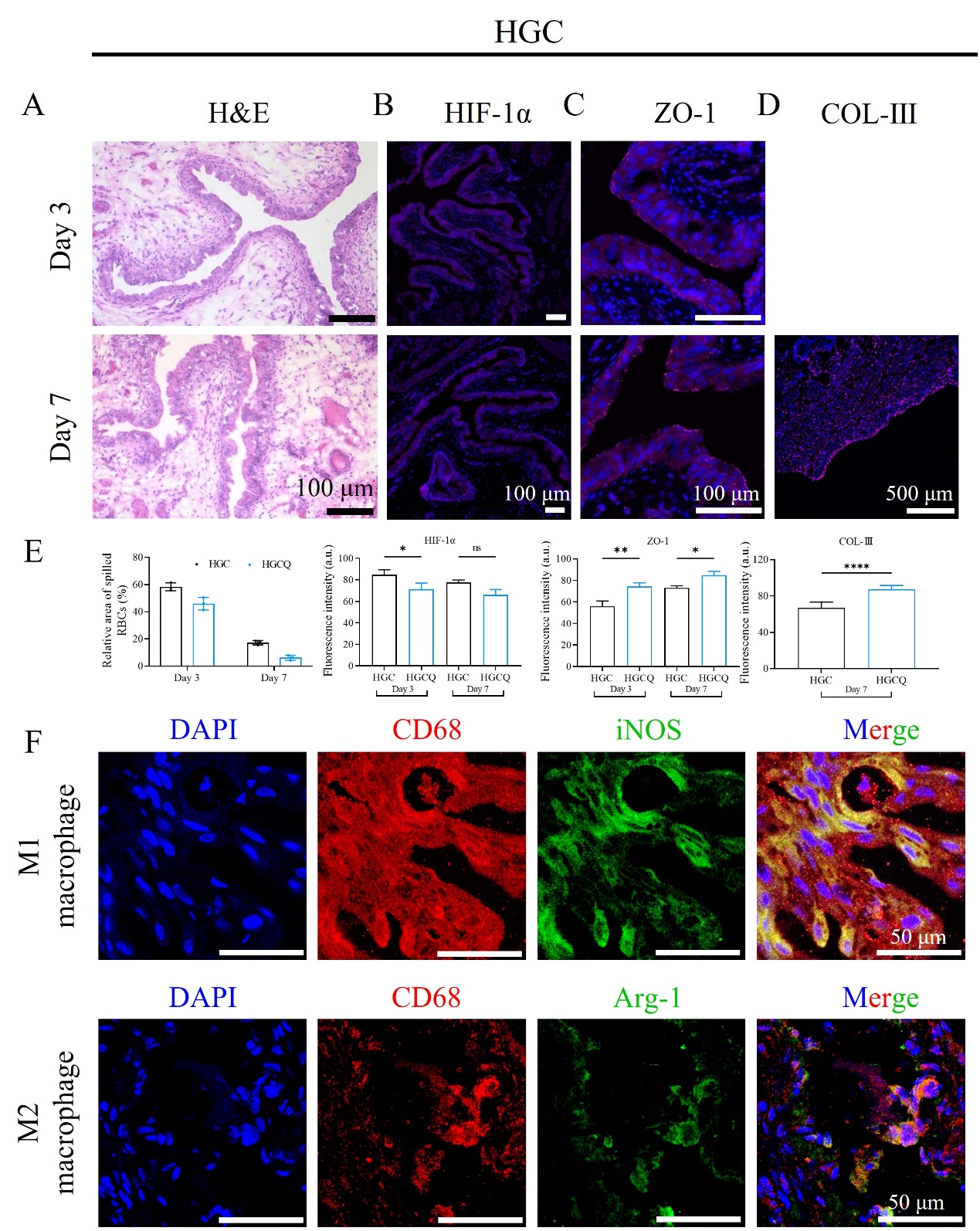


**Figure S14.** Therapeutic efficiency of HGC hydrogel in hemorrhagic cystitis treatment, (A) H&E staining of rat bladder tissues at day 3 and 7 post-treatments and Immunoﬂuorescence staining images for HIF-1α and ZO-1 after 3 and 7 days, along with COL-III staining at day 7. (B) Quantitative comparison analysis of extravasated erythrocyte infiltration areas in bladder sections Quantitative statistical analysis of HIF-1α ,ZO-1, and COL-III fluorescence. *p < 0.05, **p < 0.01, and ****p < 0.0001, n=3. (C)Immunoﬂuorescence images showing macrophages staining for M1 phenotype marker (CD68/iNOS/DAPI) and M2 phenotype markers (CD68/Arg1/DAPI) in the regenerated tissues on day 3.

| **Table S1. RT-qPCR primer sequences used in this study.** | | | | |
| --- | --- | --- | --- | --- |
| Gene | Full name | Species | Primer | Sequences(5’~3’) |
| Arg-1 | Arginase 1 | Mouse | Forward | GGCTTGCTTCGGAACTCAAC |
|  |  |  | Reverse | CATGTGGCGCATTCACAGTC |
| IL-4 | Interleukin-4 | Mouse | Forward | CCATATCCACGGATGCGACA |
|  |  |  | Reverse | AAGCACCTTGGAAGCCCTAC |
| IL-1β | Interleukin-1β | Mouse | Forward | GCAACTGTTCCTGAACTCAACT |
|  |  |  | Reverse | ATCTTTTGGGGTCCGTCAACT |
| TNF-α | Tumor necrosis factor-α | Mouse | Forward | CCCTCACACTCAGATCATCTTCT |
|  |  |  | Reverse | GCTACGACGTGGGCTACAG |
| GAPDH | Glyceraldehyde-3-phosphate | Mouse | Forward | TGGTGAAGGTCGGTGTGAAC |
|  | dehydrogenase |  | Reverse | CCATGTAGTTGAGGTCAATGAAGG |

| **Table S2. Antibodies used in this study.** | | | |
| --- | --- | --- | --- |
| Maker/Specials | Category | Dilution | Category number/ Source |
| FITC Phalloidin | - | 1:300 | CA1620**,**Solarbio,CN |
| iNOS Rabbit mAb | Primary antibody | 1:300 | AF0199,AffinityBiosciences,CN |
| CD206 Rabbit mAb | Primary antibody | 1:400 | DF4149,AffinityBiosciences,CN |
| Arg-1 Mouse mAb | Primary antibody | 1:300 | 66129-1-Ig, Proteintech, CN |
| CD68 Rabbit mAb | Primary antibody | 1:400 | 28058-1-AP,Proteintech, CN |
| HIF-1α Rabbit mAb | Primary antibody | 1:400 | GB151339-50,Servicebio,CN |
| ZO-1 Rabbit mAb | Primary antibody | 1:800 | GB111981-100,Servicebio,CN |
| COL-Ⅲ Rabbit mAb | Primary antibody | 1:1000 | GB111629-50,Servicebio,CN |
| Goat Anti-Rabbit IgG (H+L) FITC-conjugated | Secondary antibody | 1:300 | S0008,Affinity Biosciences,CN |
| Goat Anti-Rabbit IgG (H+L) CY3-conjugated | Secondary antibody | 1:300 | S0011,Affinity Biosciences,CN |
| Goat Anti-Rabbit IgG(H+L) Fluor594conjugated | Secondary antibody | 1:300 | S0006,Affinity Biosciences,CN |

**References**

[1] x. xu, Q. Feng, X. Ma, Y. Deng, K. Zhang, H. S. Ooi, B. Yang, Z.-Y. Zhang, B. Feng, L. Bian, *Biomaterials* **2022**, *289*, <https://doi.org/10.1016/j.biomaterials.2022.121802>.

[2] X. Zhang, B. Yang, L. Feng, X. Xu, C. Wang, Y. W. Lee, M. Wang, X. Lu, L. Qin, S. Lin, L. Bian, G. Li, *Bioact Mater* **2024**, *41*, 440, <https://doi.org/10.1016/j.bioactmat.2024.07.036>.

[3] Z. Tan, L. Xiao, J. Ma, K. Shi, J. Liu, F. Feng, P. Xie, Y. Dai, Q. Yuan, W. Wu, L. Rong, L. He, *Science Advances* **2024**, *10* (27), eado9120, <https://doi.org/doi:10.1126/sciadv.ado9120>.

[4] K. Wei, M. Zhu, Y. Sun, J. Xu, Q. Feng, S. Lin, T. Wu, J. Xu, F. Tian, J. Xia, G. Li, L. Bian, *Macromolecules* **2016**, *49* (3), 866, <https://doi.org/10.1021/acs.macromol.5b02527>.

[5] H. An, M. Zhang, Z. Huang, Y. Xu, S. Ji, Z. Gu, P. Zhang, Y. Wen, *Adv Mater* **2024**, *36* (8), e2310164, <https://doi.org/10.1002/adma.202310164>.

[6] Y. Chen, X. Cao, J. Yao, Z. Hu, Y. Luo, G. Li, H. Zhang, K. Wu, *Int J Biol Macromol* **2024**, *283* (Pt 4), 137487, <https://doi.org/10.1016/j.ijbiomac.2024.137487>.

[7] Y. Zhao, W. Duan, B. Zhu, Y. Chen, Y. Zhu, S. Martin‐Saldaña, Z. Xiao, X. Liu, L. Feng, Y. Ren, Y. Gong, F. Huo, J. Li, Y. Bu, B. Du, L. Zhang, *Advanced Functional Materials* **2024**, *35* (17), <https://doi.org/10.1002/adfm.202418660>.

[8] X. Ge, J. Hu, X. Qi, Y. Shi, X. Chen, Y. Xiang, H. Xu, Y. Li, Y. Zhang, J. Shen, H. Deng, *Adv Mater* **2025**, *37* (3), e2412240, <https://doi.org/10.1002/adma.202412240>.
